# Tumor-derived HMGB1 induces CD62L^dim^ neutrophil polarization and promotes lung metastasis in triple-negative breast cancer

**DOI:** 10.1038/s41389-020-00267-x

**Published:** 2020-09-17

**Authors:** Zhen Wang, Chenghui Yang, Lili Li, Xiaoyan Jin, Zhigang Zhang, Haiyan Zheng, Jun Pan, Liyun Shi, Zhou Jiang, Ke Su, Baizhou Li, Xuan Shao, Fuming Qiu, Jun Yan, Jian Huang

**Affiliations:** 1 Key Laboratory of Tumor Microenvironment and Immune Therapy of Zhejiang Province, Second Affiliated Hospital, Zhejiang University School of Medicine, Hangzhou, 310058 China; 2 Cancer Institute, Second Affiliated Hospital, Zhejiang University School of Medicine, Hangzhou, 310058 China; 3 Department of Breast Surgery, Second Affiliated Hospital, Zhejiang University School of Medicine, Hangzhou, 310058 China; 4 Department of Oncology, Second Affiliated Hospital, Zhejiang University School of Medicine, Hangzhou, 310058 China; 5 Department of Surgical Oncology, Zhejiang Taizhou Municipal Hospital, Taizhou, 318008 China; 6 Department of Gynecology, Second Affiliated Hospital, Zhejiang University School of Medicine, Hangzhou, 310058 China; 7 Department of Immunology and Medical Microbiology, Nanjing University of Chinese Medicine, Nanjing, China; 8 Department of Pathology, Second Affiliated Hospital, Zhejiang University School of Medicine, Hangzhou, 310058 China; 9 Department of Medicine and Department of Microbiology and Immunology, James Graham Brown Cancer Center, University of Louisville, Louisville, KY 40202 USA

**Keywords:** Immunosurveillance, Breast cancer

## Abstract

Triple-negative breast cancer (TNBC) is highly aggressive, difficult to treat and commonly develops visceral metastasis, including lung metastasis. We observed that High mobility group box 1 protein (HMGB1) was highly expressed in human TNBC and positively correlated with cancer metastasis. The hypoxic tumor environment is known to regulate HMGB1 secretion, but an understanding of the underlying mechanism by which tumor-derived HMGB1 regulates interstitial components and promotes breast cancer lung metastasis has remained elusive. The results of the present study showed that the number of CD62L^dim^ neutrophils, which have a strong ability to produce neutrophil extracellular traps (NETs), increased significantly in both peripheral blood and lung tissues in a mouse TNBC model and were regulated by tumor-derived HMGB1 through the TLR2 pathway. Furthermore, serum HMGB1 levels were positively correlated with CD62L^dim^ neutrophils in 86 breast cancer patients. We demonstrated that CD62L^dim^ neutrophils accelerated lung metastasis and that interventions targeting the “HMGB1-CD62L^dim^ neutrophil-NETs” axis could inhibit lung metastasis. Our results suggest that the combination of HMGB1 and CD62L^dim^ neutrophils is a potential marker for breast cancer lung metastasis and is novel target for future prevention and therapy.

## Introduction

Breast cancer is one of the most common malignant tumors worldwide. Its morbidity ranks first among women, and its mortality rate ranks third^1^. The primary cause of death in breast cancer patients is distant organ metastasis. Triple-negative breast cancer (TNBC) has the worst prognosis of breast cancers and preferentially metastasizes to the lung^2^. However, there is currently no definite marker to predict tumor metastasis.

HMGB1 is a highly conserved nuclear protein that was discovered in the calf thymus and consists of two DNA-binding HMG box domains (N-terminal A and Central B) and an acidic C-terminal tail^3^. Although there is increasing evidence of the impact of HMGB1 on tumor progression and metastasis, many contradictory findings have been reported. HMGB1 acts as a DNA chaperone in the nucleus and performs DNA repair and telomere stabilization in most cells. Furthermore, HMGB1 has been reported to bind to tumor suppressor genes (such as Rb) to regulate tumorigenesis and to promote genome stability and inhibit tumors by enhancing autophagy and maintaining telomere stability^4–6^. Interestingly, as a major damage-associated molecular pattern protein (DAMP), HMGB1 is actively released after stimulation or passively released by damaged or dying cells^7,8^. Previous studies have confirmed that HMGB1 induces the release of proinflammatory cytokines by activating proinflammatory signaling pathways^9^. At the same time, HMGB1 inhibits the antigen presentation function of dendritic cells and tumor-killing in CD8^+^ T cells, which also promotes the recruitment of M2 macrophages^10–12^. Most studies have focused on changes in HMGB1 levels in the tumor microenvironment, but its role after leaving the primary site remains unknown^3^.

Hypoxia is a hallmark of the solid tumor microenvironment, as rapid tumor growth causes a lack of oxygen and the formation of a hypoxic environment in the tumor center. Although the presence of hypoxia in tumors is independent of tumor size, grade, stage, or histological type, hypoxia regulates many pathological processes to promote tumor malignancy and is significantly associated with poor clinical outcomes in cancer patients^13^. Studies have shown that hypoxia-inducible factor-1 (HIF-1α) promotes the release of HMGB1 from cells^14^, which then form complexes with mitochondrial DNA (mtDNA) to promote tumor cell proliferation^15^.

Neutrophils are the major leukocytes in the human immune system, and their role in tumors has been underestimated due to their short lifespan and clear antibacterial ability. However, in recent years, studies have confirmed that neutrophils have prolonged lives in the presence of tumors^16^. Neutrophils play a number of roles in functional remodeling (secreting MMPs, VEGF, IL-6, and other factors) to promote tumor progression^17^. Furthermore, neutrophils play a central role in pre-metastatic niche formation and can even reactivate dormant tumor cells to promote tumor recurrence and metastasis^18,19^. The heterogeneity of neutrophils has been well shown, with identified cell types such as the tumor-killing N1 neutrophils, tumor-promoting N2 neutrophils, low-density neutrophils (LDNs) and high-density neutrophils (HDNs)^20,21^. In addition, some studies have shown that tumor cells in chronic lymphocytic leukemia promote neutrophil differentiation into the CD62L^dim^ phenotype, which exerts immunosuppressive functions^22^. However, the influence of these processes on the changes and effects on tumor metastases have not yet been reported.

In the present study, we confirmed that hypoxic primary tumors cause tumor cells to secrete HMGB1 and further promote neutrophil polarization to the CD62L^dim^ phenotype via the TLR2 signaling pathway, thereby promoting the formation of tumor metastases. Furthermore, inhibition of the HMGB1 or TLR2 signaling pathway was shown to slow tumor metastasis.

## Results

### High expression levels of HMGB1 in TNBC are associated with tumor metastasis

**Fig. 1:**
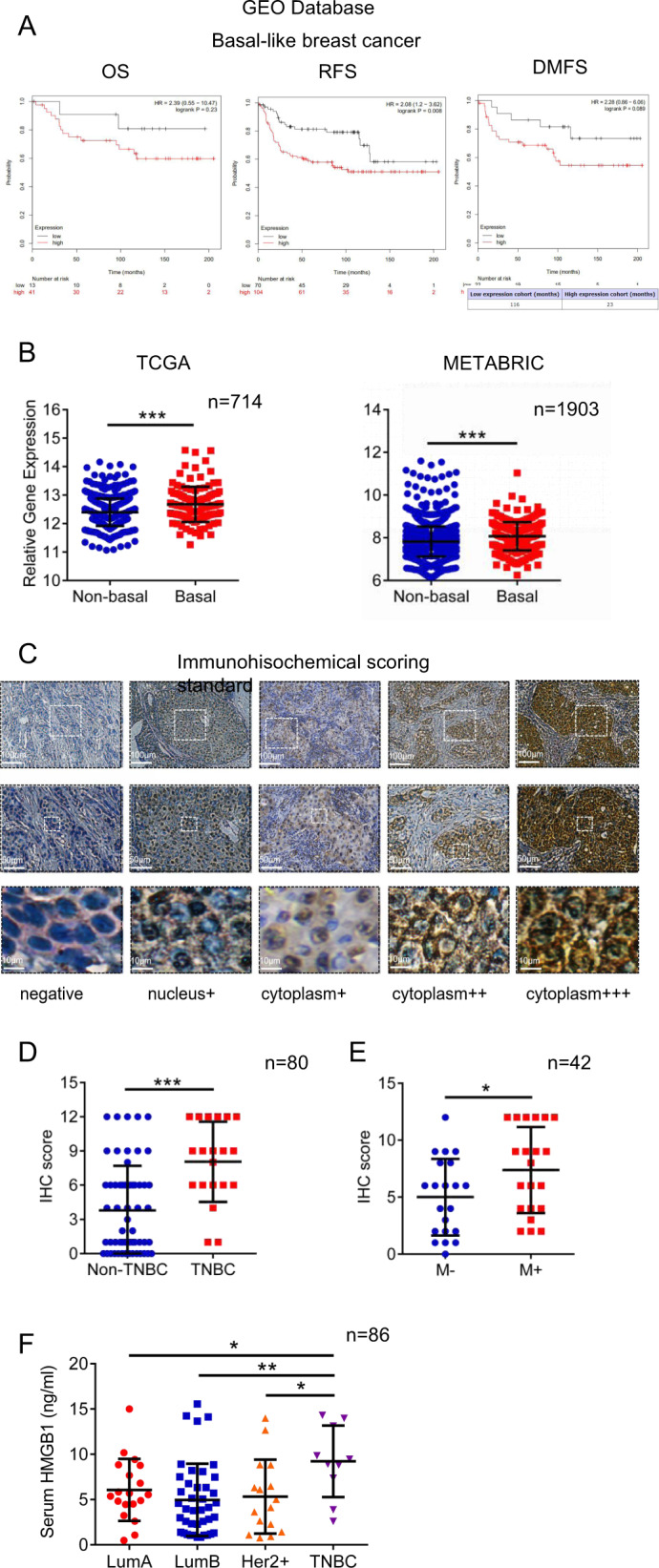
The high expression level of HMGB1 in triple-negative breast cancer patients is associated with tumor metastasis. **a** GEO database correlation analysis of HMGB1 expression with overall survival (OS), Relapse-free survival (RFS) and distance metastasis free survival (DMFS) in basal-like breast cancer patients. **b** TCGA database and METABRIC database analysis of HMGB1 expression in basal-like and non-basal-like breast cancer. **c** Representative images of HMGB1 staining scoring criteria in breast cancer. **d** The HMGB1 staining scores in triple-negative breast cancer (TNBC) and Non-TNBC. **e** The HMGB1 staining scores in primary breast cancer with and without lung metastasis for 10 years. **f** Elisa analysis of serum HMGB1 concentration in breast cancer patients with different molecular types. Data are mean ± SEM of one representative experiments. Similar results were seen in three independent experiments unless noted otherwise. Unpaired Student’s *t* tests, ns not significant. *p < 0.05, **p < 0.01, ***p < 0.001. See also Fig. S1.

The correlation between HMGB1 and the prognosis of different tumors was observed to be diverse (Fig. S1A), and the role of HMGB1 in breast cancer remains controversial^3^. According to the GEO database, among all of the patients initially diagnosed with breast cancer, patients with high HMGB1 expression are more likely to have breast cancer metastasis, especially in basal-like breast cancer (Figs. 1a and S1B). Basal-like breast cancer with increased HMGB1 expression exhibits pulmonary metastasis earlier, occurring at an average of 23 months. Further analysis of TCGA and METABRIC databases also revealed that basal-like breast cancer patients show higher levels of HMGB1 expression in primary tumors than non-basal-like breast cancer patients (Figs. 1b and S1C). Furthermore, we selected 80 pathology sections of primary breast cancer and divided them into 5 groups based on the HMGB1 expression intensity in tumor cells (Fig. 1c) and established scoring criteria (Fig. S1D). Consistent with the database results, TNBC expressed increased levels of HMGB1 (Figs. 1d and S1E), and the expression levels of HMGB1 were not correlated with tumor stage or lymph node metastasis (Fig. S1F, G). In addition, we compared breast cancer patients with lung metastasis and without tumor metastasis who underwent radical mastectomy in a span of 10 years. HMGB1 expression in the primary tumors of patients with breast cancer lung metastasis was higher than that observed in patients without tumor metastasis (Fig. 1e). In addition, we tested the serum of 86 patients and observed that the serum HMGB1 level was significantly increased in TNBC patients (Fig. 1f). These results suggest that HMGB1 is closely associated with tumor metastasis in breast cancer, especially in TNBC.

### Tumor cell-derived HMGB1 is responsible for lung metastasis of breast cancer

**Fig. 2:**
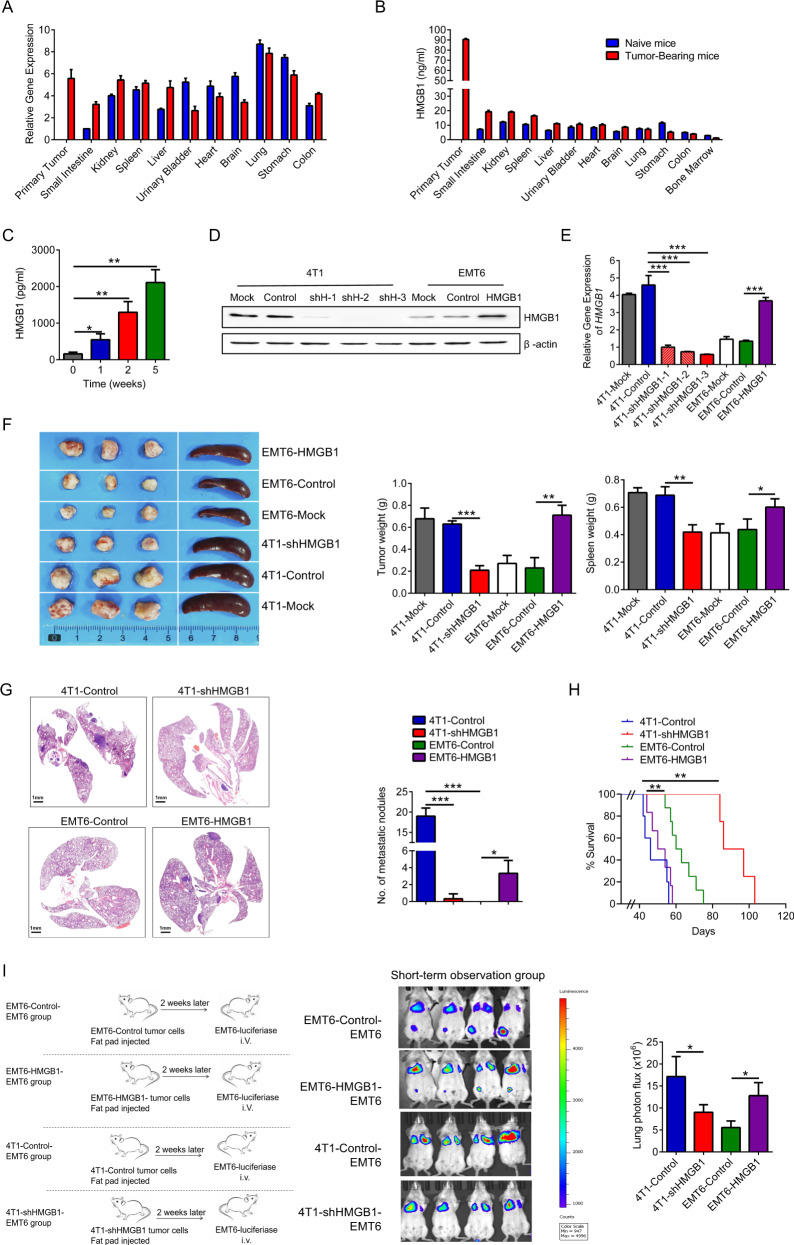
HMGB1 derived from tumor cells is responsible for lung metastasis of breast cancer. **a** mRNA expression of HMGB1 in various organs of naive mice and 2-week tumor-bearing mice. **b** Elisa analysis of HMGB1 concentration in cytoplasm of various organs of naive mice and 2-week tumor-bearing mice. **c** Elisa analysis of serum HMGB1 concentration with tumor progression. **d**, **e** The protein level (**d**) and the mRNA level (**e**) of HMGB1 in different tumor cell lines. 4T1-shHMGB1, *HMGB1* knockout 4T1 cell line; EMT6-HMGB1, *HMGB1* overexpressing EMT6 cell line. **f** Representative image of primary tumors and spleens of 4-week tumor-bearing mice formed by different tumor cell lines inoculation. **g** H&E-stained lung sections (left) and quantification (right) of lung metastasis of 4-week tumor-bearing mice. **h** Survival of mice (*n* = 10 each) after different tumor cell lines inoculation (Kaplan–Meier test). **i** Schematic illustration (left) and representative bioluminescent imaging (middle) and lung bioluminescent quantification (right) of Balb/c mice injected with luciferase-expressing EMT6 cells observed in 2 h. Data are mean ± SEM of one representative experiments. Similar results were seen in three independent experiments. Unpaired Student’s *t* tests, ns not significant. *p < 0.05, **p < 0.01, ***p < 0.001. See also Fig. S2.

To elucidate the role of HMGB1 in breast cancer, we used a 4T1 orthotopic mouse model of TNBC. As a conserved nucleoprotein, HMGB1 is typically expressed in mammalian cells and is widely distributed in various organs (Fig. 2a). Accumulating evidence has demonstrated that HMGB1, as a secreted protein, performs functions other than maintaining DNA stability^23^. Therefore, HMGB1 secretion levels in various organs were assessed, and the primary tumors exhibited the highest HMGB1 levels (Fig. 2b). HMGB1 expression in primary tumors increased with tumor progression (Fig. S2A, B), and the concentration of HMGB1 in serum also increased over time (Fig. 2c). The composition of primary tumors is complex, and a variety of cells are involved in the secretion of HMGB1^3^. Both immune cells and tumor cells in primary tumors expressed high levels of HMGB1 (Fig. S2C). However, the primary source of HMGB1 in tumor tissue culture supernatant (TTCS) was tumor cells rather than immune cells (Fig. S2D, E). EMT6 is a tumor cell line with low lung metastatic ability^24^. Compared to 4T1 cells, EMT6 cells showed reduced HMGB1 expression (Fig. 2d, e). Therefore, we constructed the cell lines 4T1-shHMGB1 and EMT6-HMGB1, which have silenced or overexpressed HMGB1, respectively. The transfection dose did not affect the proliferation of these cell in vitro (Fig. S2F). Mice were orthotopically inoculated with the constructed tumor cells. After 2 weeks, no significant difference was observed between tumor and spleen size (Fig. S2G, H). However, with tumor progression, high HMGB1 expression was observed to promote tumor growth in both the 4T1 and EMT6 tumors (Fig. 2f), and lung metastasis was obvious in the 4T1-Control and EMT6-HMGB1 groups but not the 4T1-shHMGB1 and EMT6-Control groups at 4 weeks (Fig. 2g). The survival time of the 4T1-shHMGB1 group was significantly longer than that of the 4T1-Control group, while the life span of the EMT6-HMGB1 group was shortened (Fig. 2h). To exclude the effect of tumor burden on tumor metastasis, we conducted a study at the early tumor-bearing stage in mice with insignificant tumor differences. The mice in the 4T1-Control and EMT6-HMGB1 groups had high HMGB1 expression in primary tumors and serum (Fig. S2I, L). In the lung tissue of mice, pre-metastatic- associated gene expression was significantly decreased in the 4T1-shHMGB1 and EMT6-Control groups (Fig. S2M), indicating that HMGB1 contributes to pre-metastatic niche formation. To further understand the correlation between the formation of the pre-metastatic niche and tumor metastasis, we established different short-term observation mouse tumor models (Fig. 2i). In the mice that received the non-metastatic breast cancer EMT6-Control-EMT6 group, the imaging results showed that EMT6-luciferase tumor cells (i.v.) returned to the primary tumor site but not lung. Moreover, HMGB1 overexpression in EMT6 cells (in situ) can cause more EMT6-luciferase tumor cells (i.v.) to remain in lung tissue. Compared to the 4T1-Control-EMT6 group, more EMT6-luciferase cells (i.v.) accumulated in the lung than was observed in the 4T1-shHMGB1-EMT6 group. Taken together, these data suggest that HMGB1 secreted by the primary tumor plays an important role in lung pre-metastatic niche formation.

### Hypoxia regulates the release of HMGB1 from the nucleus to the cytoplasm

**Fig. 3:**
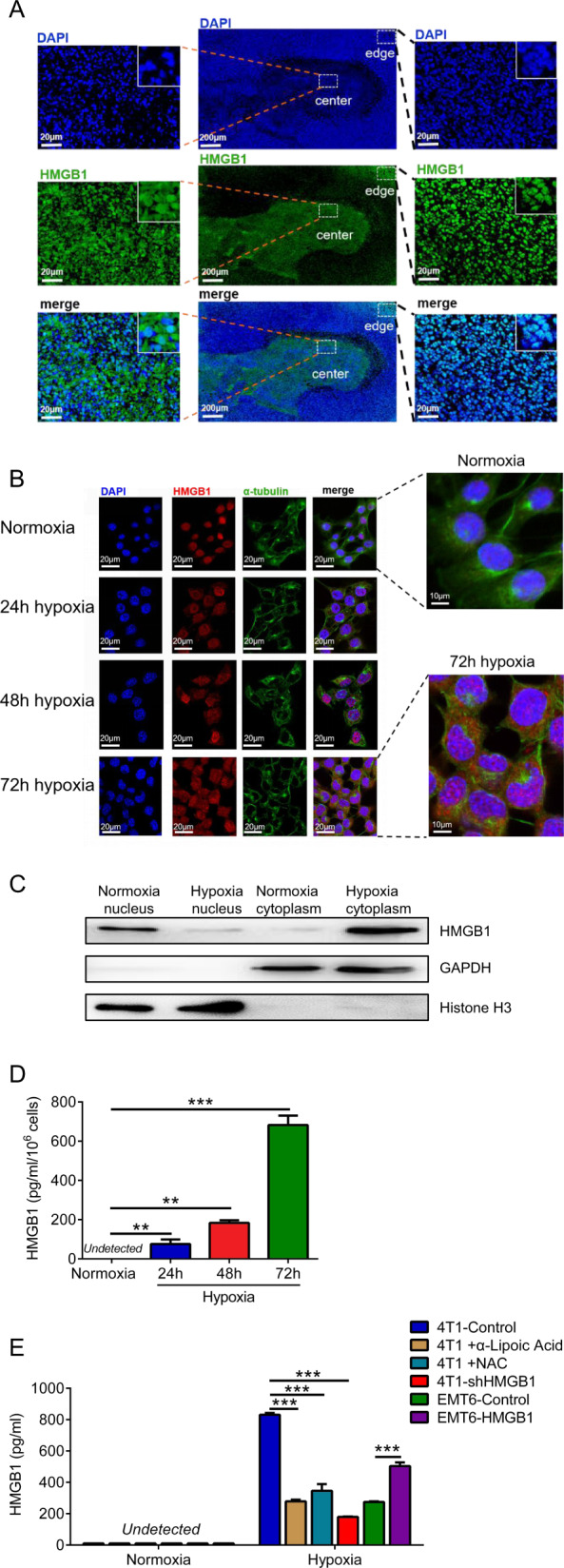
Hypoxia regulates the release of HMGB1 from the nucleus into the cytoplasm. **a** Representative images on HMGB1-stained primary tumor sections of 2-week tumor-bearing mice. **b** Cell immunofluorescent analysis of HMGB1 localization in 4T1 tumor cells cultured with normoxia or hypoxia for 24, 48, and 72 h, respectively. Cells were stained with HMGB1 (red), α-tubulin (green) and DAPI(blue). **c** Western blot analysis of HMGB1 in cytoplasmic and nuclear components of tumor cell lines under normoxic and hypoxic conditions. **d** Elisa analysis of HMGB1 concentration of 4T1 tumor cells cultured supernatant (TCCS) under normoxic and varying degrees of hypoxic conditions. **e** Elisa analysis of HMGB1 concentration of different TCCS under normoxic and hypoxic (72 h) conditions treated with or without α-lipoic acid and N-acetyl-L-cysteine (NAC). Data are mean ± SEM of one representative experiments. Similar results were seen in three independent experiments. Unpaired Student’s *t* tests, ns not significant. *p < 0.05, **p < 0.01, ***p < 0.001. See also Fig. S3.

As an important DAMP, HMGB1 enters the cytoplasm from the nucleus when cells are subjected to abnormal stimulation^25^. Oxygen is one of the primary reasons for HMGB1 localization changes, and the local characteristics of tumors include low oxygen and low pH conditions^15^. In the hypoxic tumor center, HMGB1 was located in the cytoplasm of tumor cells, whereas in the normoxic tumor edge it was present in the nucleus of tumor cells (Figs. S3A and 3a). HMGB1 entered the cytoplasm of 4T1 cells from the nucleus under hypoxia for 72 h (Fig. 3b). The effect of hypoxia on HMGB1 was not reflected in the changes in transcription or protein levels (Fig. S3B, C) but rather in the translocation from the nucleus to the cytoplasm (Fig. 3c). The concentration of HMGB1 in the tumor cell culture supernatant (TCCS) increased gradually with the prolongation of hypoxia (Fig. 3d). Moreover, hypoxia did not cause the death of tumor cells, either 4T1 or EMT6, indicating that HMGB1 is the product of cell secretion rather than cell lysis (Fig. S3D). Compared to the 4T1-shHMGB1 group, the 4T1-Control group showed increased HMGB1 expression under hypoxic conditions, which could be reversed by treatment with antioxidants such as α-lipoic acid and N-Acetyl-L-cysteine (NAC) (Fig. 3e). In addition, HMGB1 overexpression could also increase the secretion by EMT6 under hypoxia. Here we show that the release of HMGB1 is controlled by hypoxia.

### Hypoxia-induced release of HMGB1 from primary tumors polarizes neutrophils into CD62L^dim^ neutrophils

**Fig. 4:**
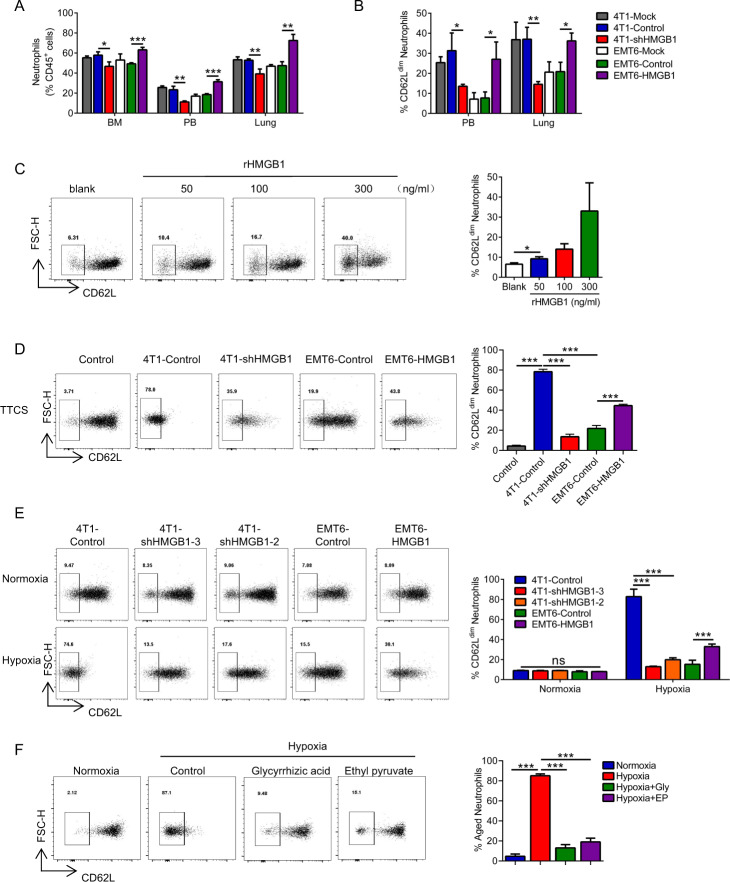
Hypoxia-released HMGB1 from primary tumor induce neutrophils to CD62L^dim^. **a** Quantification of neutrophils in BM, PB, and lung of 2-week tumor-bearing mice formed by different tumor cell lines inoculation. **b** Quantification of CD62L^dim^ neutrophils in PB and lung infiltrating neutrophils of 2-week tumor-bearing mice formed by different tumor cell lines inoculation. **c** Quantification of CD62L expression on neutrophils from BM neutrophils of naive mice treated with rHMGB1 for 4 h in vitro. **d** Flow analysis of CD62L^dim^ neutrophils in BM neutrophils of naive mice stimulated with different concentrated tumor tissue culture supernatant (TTCS) of tumor-bearing mice. **e** Flow analysis proportions of CD62L^dim^ neutrophils in BM neutrophils of naive mice stimulated with different concentrated TCCS under normoxic and hypoxic (72 h) conditions. **f** Flow analysis proportions of CD62L^dim^ neutrophils from naive mice BM induced with concentrated TCCS from normoxic or hypoxic (72 h) tumor cell with or without glycyrrhizic acid and ethyl pyruvate. Data are mean ± SEM of one representative experiments. Similar results were seen in three independent experiments. Unpaired Student’s *t* tests, ns, not significant. *p < 0.05, **p < 0.01, ***p < 0.001. See also Fig. S4.

Neutrophils of tumor-bearing mice with different tumor cells were examined. Neutrophils in the 4T1-shHMGB1 groups had significantly reduced bone marrow mobilization, peripheral blood distribution, and lung tissue infiltration than those from the 4T1-Control group, and HMGB1 overexpression promoted these conditions in the EMT6 groups (Figs. 4a and S4A, B). These results were consistent with the previously observed ability of HMGB1 to mobilize neutrophils^26^. Importantly, the number of CD62L^dim^ neutrophils was significantly increased in the 4T1-Conrtrol and EMT6-HMGB1 groups compared with that in the 4T1-shHMGB1 and EMT6-Control groups (Figs. 4b and S4C). This result suggests that CD62L^dim^ neutrophils have an important function in pre-metastatic niche formation and that HMGB1 is crucial for the activation of neutrophils. Furthermore, we observed that recombinant HMGB1 (rHMGB1) can directly induce neutrophil polarization toward the CD62L^dim^ phenotype (Fig. 4c). The tumor tissue cultured supernatant (TTCS) from the 4T1-Control and EMT6-HMGB1 groups had the strongest ability to induce CD62L^dim^ neutrophils, while TTCS from the 4T1-shHMGB1 and EMT6-Control groups had comparatively weaker effects (Fig. 4d). In vitro cultured of tumor tissue for 24 h did not lead to tumor cell death (Fig. S4D). Moreover, only the hypoxic TCCS of the 4T1-Control and EMT6-HMGB1 groups effectively induced CD62L^dim^ neutrophils (Fig. 4e). This effect was inhibited by neutralizing HMGB1 (with glycyrrhizic acid) or inhibiting the release of HMGB1 from the cytoplasm (with ethyl pyruvate) (Fig. 4f). These results suggest that HMGB1 from tumor cells has an important impact on CD62L^dim^ neutrophils.

### HMGB1 polarizes neutrophils to CD62L^dim^ neutrophils in breast cancer patients

**Fig. 5:**
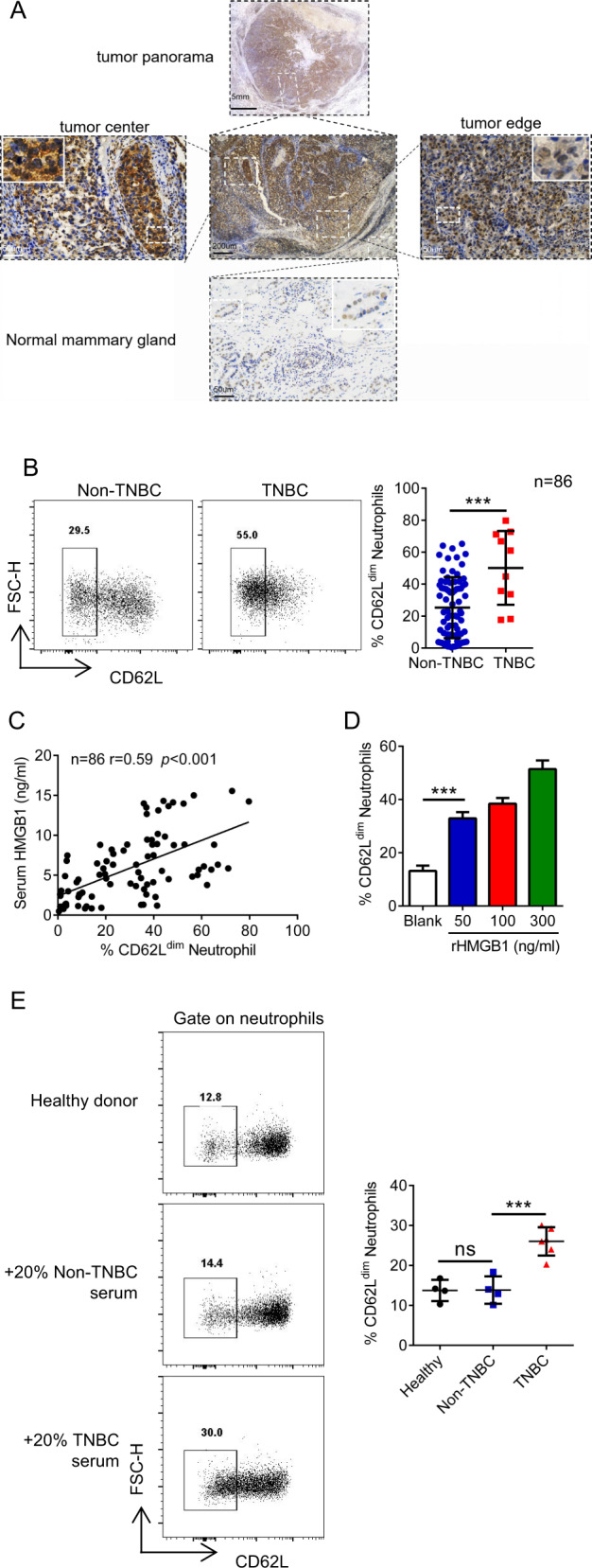
HMGB1 induces neutrophils to CD62L^dim^ neutrophils in breast cancer patients. **a** Representative images of HMGB1 staining in TNBC. **b** Flow-cytometry gate strategy (left) and analysis proportions (right) of human PB CD62L^dim^ neutrophils detection. **c** The Pearson correlation between serum HMGB1 expression (by Elisa) and paired PB CD62L^dim^ neutrophils proportion of breast cancer patients (by Flow). **d** Change of CD62L expression on neutrophils from human PB treated with rHMGB1 for 4 h in vitro. **e** Change of CD62L expression on neutrophils from healthy donor PB treated with different patients’ serum. Data are mean ± SEM of one representative experiments. Similar results were seen in three independent experiments. Unpaired Student’s *t* tests, ns, not significant. *p < 0.05, **p < 0.01, ***p < 0.001. See also Fig. S4.

In the tumor sections of TNBC patients, in the tumor center, HMGB1 was located in the cytoplasm, while in the tumor margin, HMGB1 was located in the nucleus. However, HMGB1 was not detected in peritumoral gland duct cells (Fig. 5a). We assessed the peripheral blood (PB) neutrophil proportion and the paired serum HMGB1 level of newly diagnosed breast cancer patients and observed that TNBC patient PB had a higher proportion of CD62L^dim^ neutrophils than that from Non-TNBC patients (Figs. 5b and S4E). In addition, serum HMGB1 expression levels were positively correlated with the proportion of PB CD62L^dim^ neutrophils (Fig. 5c). Furthermore, rHMGB1 induced human CD62L^dim^ neutrophils in a dose-dependent manner (Fig. 5d). We treated the neutrophils from healthy donors with different patient sera and observed that the TNBC serum of patients had a stronger ability to induce CD62L^dim^ neutrophil polarization (Fig. 5e). These results further support the hypothesis of a “tumor-secreted HMGB1-CD62L^dim^ neutrophil” axis in human breast cancer patients.

### CD62L^dim^ neutrophils are induced by HMGB1 via TLR2 to form NETs

**Fig. 6:**
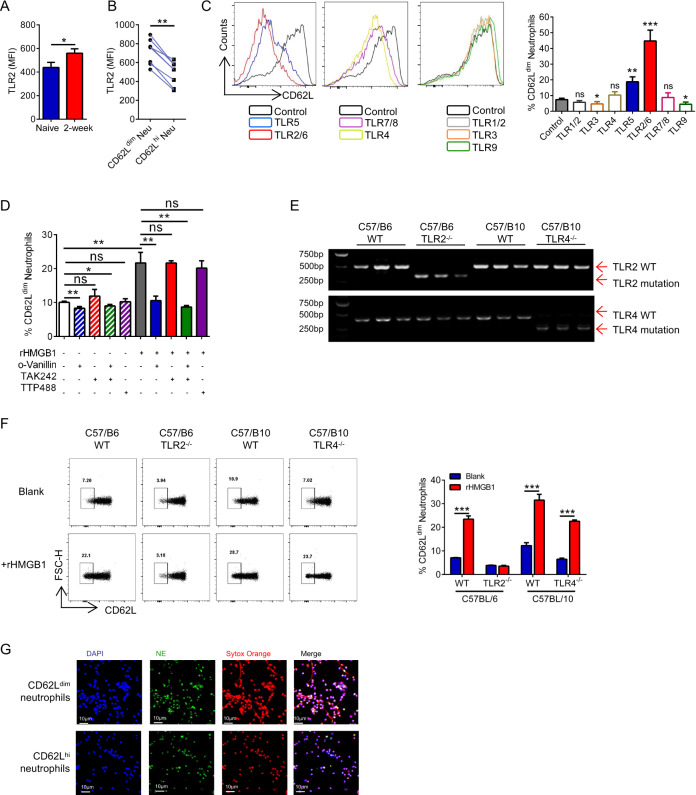
CD62L^dim^ neutrophils are induced via TLR2 by HMGB1 to form NETs. **a** MFI of TLR2 expression on lung infiltrating neutrophils of naive mice and tumor-bearing mice by flow. **b** MFI of TLR2 expression on lung infiltrating CD62L^dim^ and CD62L^hi^ neutrophils of tumor-bearing mice by flow. **c** Flow analysis (left) and the quantification (right) of CD62L expression on neutrophils from naive mice BM treated with various TLR agonists, which included Pam3CSK4, FSL-1, Poly(I:C), MPLA-SM, FLA-ST, R848, and TLR9 Agonist Kit. **d** Purified BM neutrophils of naive mice were pre-treated with O-Vanillin (TLR2 inhibitor) and TAK242 (TLR4 inhibitor), TTP488 (RAGE inhibitor) followed by stimulation with rHMGB1. Proportions of CD62L^dim^ neutrophils were analyzed by flow. **e** Gel electrophoresis patterns of TLR2^−/−^ and TLR4^−/−^ mice. **f** Quantification of CD62L expression on neutrophils from BM neutrophils of different naive mice treated with rHMGB1 for 4 h in vitro. **g** Cell immunofluorescent analysis of neutrophils extracellular traps (NETs) of lung infiltrating CD62L^dim^ neutrophils and CD62L^hi^ neutrophils of 2-week tumor-bearing mice. NETs staining according to NE (green), Cytox Orange (red) and DAPI (blue). Data are mean ± SEM of one representative experiments. Similar results were seen in three independent experiments. Unpaired Student’s *t* tests, ns not significant. *p < 0.05, **p < 0.01, ***p < 0.001. See also Fig. S5.

The morphology and development of neutrophils are greatly influenced by the activities of Toll-like receptors (TLRs)^27^. HMGB1 receptors primarily include TLR2, TLR4, and receptor for advanced glycation end products (RAGE)^25,28,29^. Elevated TLR2 and TLR4 levels were observed in lung neutrophils from tumor-bearing mice (Figs. 6a and S5A). In addition, CD62L^dim^ neutrophils expressed higher levels of TLR2 and MyD88 than CD62L^hi^ neutrophils (Figs. 6b and S5B), but there was no significant difference in TLR4 levels (Fig. S5C). However, FLA-ST (TLR5 agonist) and FSL-1 (TLR2/6 agonist) induced CD62L^dim^ neutrophils (Fig. 6c), while D-ribose (RAGE agonist) did not affect the expression of CD62L on neutrophils (Fig. S5D). Bacterial flagellin is the only ligand recognized by TLR5^30^. TLR2 works as a part of TLR1/2 and TLR2/6 complexes^31^, whereas CD62L^dim^ neutrophils are induced by activation of the TLR2/6 complex. Although the downstream target of TLR2 and TLR4 is MyD88, their effects were not completely consistent. Only o-Vanillin (TLR2 inhibitor) effectively inhibited the induction of CD62L^dim^ neutrophils by rHMGB1 (Fig. 6d). To further assess the role of TLR2 and TLR4 in CD62L^dim^ neutrophils, *TLR2*^−*/*−^ and *TLR4*^−/−^ transgenic mice were used (Fig. 6e), and the results showed that rHMGB1 did not induce *TLR2*^*−/−*^ neutrophils into CD62L^dim^ neutrophils (Fig. 6f). NETs formation is an important property of neutrophils, and a growing amount of evidence indicates a role for NETs not only in infections but also in noninfectious inflammatory diseases, including cancer^32^. CD62L^dim^ neutrophils exhibited a stronger ability to form NETs than CD62L^hi^ neutrophils (Fig. 6g). In addition, the FSL-1 and rHMGB1 also induced NETs formation in neutrophils (Fig. S5E), while *TLR2*^*−/−*^ neutrophils could not form effective NETs when induced by rHMGB1 (Fig. S5F). The results suggest that the effect of HMGB1 on CD62L^dim^ neutrophils may be mediated by TLR2.

### Lung metastasis in breast cancer is mediated by the “HMGB1-TLR2-CD62L^dim^ neutrophil-NETs” axis

**Fig. 7:**
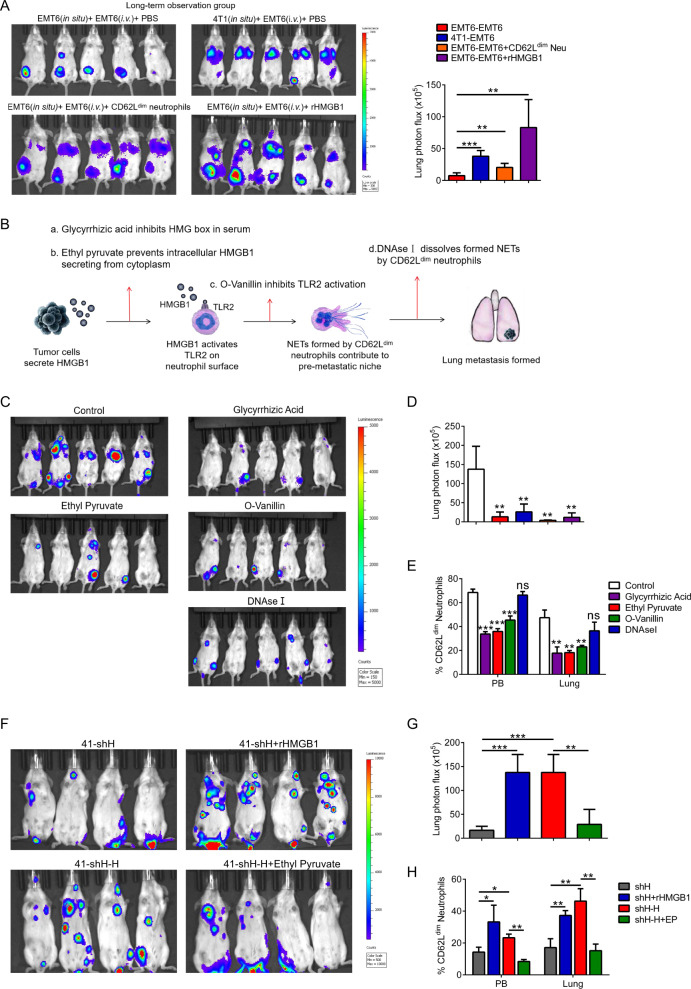
“HMGB1-TLR2-CD62L^dim^ neutrophils-NETs” axis is responsible for lung metastasis of breast cancer. **a** Representative bioluminescent imaging (left) and lung bioluminescent quantification (right) of Balb/c mice injected with luciferase-expressing EMT6 cells after CD62L^low^ neutrophils or rHMGB1 reinjection for 2 weeks. **b**, **d** Schematic illustration (**b**), representative bioluminescent imaging (**c**) and lung bioluminescent quantification (**d**) of Balb/c mice injected with luciferase-expressing 4T1 cells after multiple interventionrs for 2 weeks. **e** Flow analysis of PB and lung infiltrating CD62L^dim^ neutrophils in tumor-bearing mice after multiple intervention for 2 weeks. **f**, **g** Representative bioluminescent imaging (**f**) and lung bioluminescent quantification (**g**) for 4T1-shHMGB1 tumor-bearing mice in rescue experiments. 4T1-shH,4T1-shHMGB1; 4T1-shH-H, *HMGB1* overexpressing 4T1-shHMGB1. **h** Flow analysis of PB and lung infiltrating CD62L^dim^ neutrophils in for 4T1-shHMGB1 tumor-bearing mice in rescue experiments. EP, Ethyl Pyruvate. Data are mean ± SEM of one representative experiments. Similar results were seen in three independent experiments. Unpaired Student’s *t* tests, ns not significant. *p < 0.05, **p < 0.01, ***p < 0.001. See also Fig. S6.

To assess the ability of CD62L^dim^ neutrophils to promote tumor metastasis, we also established different long-term observation groups (Fig. S6A). The in situ injection of 4T1 cells significantly increased the growth of EMT6-luciferase cells (i.v.) in the lung (Fig. 7a). Continuous injection of rHMGB1 or CD62L^dim^ neutrophils also increased the formation of metastasis of EMT6-luciferase (i.v.) in the lung. In addition, we also pretreated mice with rHMGB1 and then inoculated them with 4T1 cells in situ, observing that rHMGB1 also promoted the lung metastasis of tumor cells in advance (Fig. S6B). Furthermore, different interventions were administered to the tumor metastasis model (Fig. 7b), including inhibiting HMGB1 secretion from the cytoplasm, inhibiting the function of the HMG box of HMGB1, inhibiting the activation of TLR2, or clearance of the formed NETs. After 2 weeks of treatment, lung metastasis of the tumor was reduced to different degrees (Fig. 7c, d). Moreover, we tested the PB and lung CD62L^dim^ neutrophils from the different groups of mice and observed that inhibiting HMGB1 by glycyrrhizic acid or ethyl pyruvate and blocking TLR2 activation by O-Vanillin reduced the proportion of CD62L^dim^ neutrophils, while DNase I had no effect on the frequency of CD62L^dim^ neutrophils (Fig. 7e). In vitro immunofluorescence results confirmed that DNase I effectively reduced the formation of NETs (Fig. S6C). Furthermore, we conducted the rescue experiment for the 4T1-shHMGB1 group (Fig. S6D, E). Metastasis was observed at 4 weeks (Fig. 7f, g) and CD62L^dim^ neutrophils were detected under different interventions (Fig. 7h). These results again indicated that HMGB1 may promote lung metastasis of breast cancer through TLR2-CD62L^dim^ neutrophils.

## Discussion

L-Selectin (CD62L) is a type I transmembrane cell adhesion molecule that is expressed on most leukocytes, and numerous studies have shown that L-selectin downstream signaling can affect the movement of neutrophils, such as rolling, adhesion and migration^33,34^. Subsequently, CD62L was shown to not only affect the behavior of neutrophils but also regulate their activation^35^. CD62L^dim^ neutrophils are thought to be an independent subset due to their unique transcriptional properties in response to acute inflammation^36^, while another study illustrated their poor antimicrobial activity^37^. However, scholars in different fields have reported that CD62L^dim^ neutrophils present as an immunosuppressive subset that promotes disease progression, especially in chronic lymphocytic leukemia^22,38,39^. In contrast, the literature suggests that CD62L^dim^ neutrophils are associated with improved survival in patients with head and neck squamous cell carcinoma (HNSCC)^40^. These results suggest that the role of this subset is not uniform in different backgrounds. The role of CD62L^dim^ neutrophils in breast cancer, especially metastatic breast cancer, has not been reported. We propose, for the first time, that CD62L^dim^ neutrophils are significantly increased during the early stage of tumor progression and are regulated by HMGB1 secreted from the primary tumor. Persistent transfusion of CD62L^dim^ neutrophils promotes metastasis and even lung metastasis of low-metastatic-potential EMT6 tumor cells.

A previous study reported that HMGB1 recruits neutrophils to promote melanoma progression^28^. We observed that HMGB1 produced by hypoxic tumors induced neutrophils to lose CD62L. Tumor tissues formed by HMGB1 knockdown tumor cells exhibited a reduced proportion of CD62L^dim^ neutrophils and reduced lung metastasis of tumors, effectively extending the survival period of the mice. We further demonstrated that HMGB1 overexpression partially increased the metastasis capacity of EMT6, and more importantly, significantly increased the number of CD62L^dim^ neutrophils in the EMT6-HMGB1 group. To further support our findings, data from database and our primary human tumor specimens showed that TNBC patients express higher levels of HMGB1 than non-TNBC patients. Moreover, breast cancer patients with lung metastasis were shown to express higher levels of HMGB1 than 10-year metastasis-free patients. In addition, patient serum HMGB1 had a significant positive correlation with the proportion of PB CD62L^dim^ neutrophils. Therefore, the induction of CD62L^dim^ neutrophil production by high levels of HMGB1 may accelerate lung metastasis of TNBC.

HMGB1 promotes CD62L^dim^ neutrophils to release NETs through the TLR2 signaling pathway. Previous studies have suggested that NETs possess antitumor effects^41^. However, recent studies have suggested that NETs promote cancer metastasis^42,43^. We demonstrate that CD62L^dim^ neutrophils have robust NETs formation ability and that clearing NETs by DNase I reduces tumor lung metastasis. Taken together, these findings suggest that CD62L^dim^ neutrophils accelerate lung metastasis by enhancing NET formation.

The prevention and treatment of cancer metastasis is the ultimate goal of cancer research. It is not wise to remove all neutrophils in vivo due to the important immunosurveillance role of the immune system and the serious consequences of neutropenia. In vivo interventions targeting CD62L^dim^ neutrophils and their related pathways reduce tumor lung metastasis. In this study, we showed that CD62L^dim^ neutrophils are a neutrophil subset with clear markers and are a promising intervention target.

In summary, in the present study, we demonstrated that HMGB1 secreted by hypoxic tumor cells induces CD62L^dim^ neutrophils, thereby promoting lung metastasis via enhanced NETs formation. These findings suggest that CD62L^dim^ neutrophils and HMGB1 are potential targets to prevent breast cancer lung metastasis. We believe that the expression level of tumor tissue or serum HMGB1 combined with the PB CD62L^dim^ neutrophil percentage may be a prospective strategy to predict the possibility of tumor metastasis in breast cancer patients.

## Materials and methods

### Human samples

Peripheral blood (PB) and tissue paraffin sections were collected from the Second Affiliated Hospital of Zhejiang University, School of Medicine. Clinico-pathological characteristics of patients included in this study were shown in Supplementary Tables 1–3. Blood samples were collected by licensed practical nurses at 6:00 a.m. before any treated intervention. Blood cells from Sodium Heparin Blood Collection Tube (BD vacuum™) were lysed for 15 min (BD Biosciences, #349202) and washed for subsequent flow cytometry analysis. Serum from Rapid Serum Tube (BD Vacutainer®) was obtained after 3000 rpm, 5 min centrifugation, and stored for ELISA test. All patients signed the informed consent, which was approved by the ethics review committee of the Second Affiliated Hospital of Zhejiang University School of Medicine.

### Data obtain and analysis from database

Survival data of breast cancer and pan-tumor were obtained from Kaplan–Meier plotter (http://kmplot.com/analysis/). The Cancer Genome Atlas (TCGA) breast cancer patient cohort data were downloaded from the TCGA website (https://cancergenome.nih.gov), and the METABRIC cohort data was downloaded from the cBioPortal website (https://www.cbioportal.org). 714 cases from TCGA and 1903 cases from METABRIC were enrolled. Data classification and analysis was operated by R software (V4.0.1).

### Cell lines

4T1 cell lines were purchased from Shanghai Institute of Cell Biology of the Chinese Academy of Science (SIBS, Shanghai, China). EMT6 cell lines were purchased from FuDan IBS Cell Center (FDCC, Shanghai, China). Both cell lines were authenticated by STR profiling. Cells were cultured in RPMI-1640 medium containing 10% FBS (Gibco), 2 mmol/L glutamine (Sigma-Aldrich) and 1:100 penicillin-streptomycin (Gibco). All of them were incubated in a humidified incubator at 37 °C supplied with 5% CO_2_.

To culture in hypoxic condition, tumor cells were plated and put in hypoxic incubator (1% oxygen) after cells attached. Two antioxidants including α-lipoic acid (1 mM, Solarbio, #IL0180) and N-acetyl-L-cysteine (1 mM, Solarbio, #C8460) were added (Fig. 3e).

### HMGB1 short hairpin RNA (shRNA) and overexpression stable cell lines

4T1 cells were infected with shHMGB1 lentivirus or non-silencing control for 48 h. Stable clones were selected using puromycin (5 μg/mL). The HMGB1 shRNA oligonucleotide sequences were as follows: HMGB1 shRNA1: 5′-CCGGGAAGATGATGATGATGAATAATCTCGAGATTATTCATCATCATCATCTTCTTTTTG-3′. HMGB1 shRNA2: 5′-CCGGTTGGTGCACAGCACAAATTAGCTCGAGCTAATTTGTGCTGTGCACCAATTTTTG-3′. HMGB1 shRNA3: 5′-CCGGATGCAGCTTATAGAAGATAACTCGAGTTATCTTCGTATAAGCTGCATTTTTTG-3′. Non-silencing shRNA (control shRNA) were used as mock-transfected controls. Then HMGB1 expression was verified by real-time-PCR and western blot.

4T1-shHMGB1 and EMT6 cells were infected with lentivirus which overexpressed HMGB1 (NCBI reference sequence ID, NM_001313894.1) or its control for 48 h. Stable clones were selected using Blasticidin S (5 μg/mL).

### Mice

Wild-type BALB/c female mice were purchased from Slaccas (Shanghai, China). TLR2^−/−^ C57BL/10 female mice were kindly gifted from Dr. Deborah A. Quinn (Massachusetts General Hospital, Harvard Medical School). TLR4^−/−^ C57BL/6, Wild-type C57BL/6, and C57BL/10 female mice were purchased from Gempharmatech (Nanjing, China).

Genotyping of knockout mice were operated as protocol provided from Dr. Deborah A. Quinn and Gempharmatech. Briefly, mouse toes were ground with liquid nitrogen, and DNA was obtained using Animal Genomic DNA Isolation Kit (Sangon Biotech, #B518221), and further amplified by PrimerSTAR Max DNA Polymerase (TaKaRa, #R045A). Amplified DNA was then detected by Agarose Gel Electrophoresis on a 1.5% agarose gel (Sigma-Aldrich, #A9539). For TLR2^−/−^ mice genotyping, primers were used as follows: (Common) 5′-CTTCCTGAATTTGTCCAGTACA-3′; (Mutant, 334 bp) 5’-GGGCCAGCTCATTCCTCCCAC-3’; (Wild Type, 499 bp) 5′-ACGAGCAAGATCAACAGGAGA-3′. For TLR4^−/−^ mice genotyping, primers were used as follows: TLR4 mutant (140 bp): (Forward) 5′-GCAAGTTTCTATATGCATTCTC-3′, (Reverse) 5’-CCTCCATTTCCAATAGGTAG-3’; TLR4 wild type (390 bp): (Forward) 5’-ATATGCATGATCAACACCACAG-3’, (Reverse) 5′-TTTCCATTGCTGCCCTATAG-3′. Six- to eight-week old age-matched mice were randomly assigned to different groups. BALB/c mice were anesthetized with 0.8% pentobarbital intraperitoneal (i.p.) injection and 100 μl cell suspension (4T1 or EMT6, 1 × 10^6^ cells /mL) were implanted in the right fourth mammary fat pad. All animal procedures are approved by Ethic Review Committee of the Second Affiliated Hospital of Zhejiang University School of Medicine.

### Specimen acquisition and processing

All mice specimens were processed at 9:00 a.m. to prevent the effects of circadian rhythm. Bone marrow (BM) cells from the hind limbs of mice were extracted and filtered. PB was taken from the eyeball and placed in the heparin tube, then lysed for 15 min. The primary tumor and lung tissue were cut into small pieces and digested in medium containing 1 mg/mL collagenase IV (Sigma-Aldrich, #V900893) in 37 °C shaking incubator for 2 h. Cell suspension was then filtered through 40 μm Nylon mesh (BD FALCON, #352340) for subsequent testing or culture in vitro.

### Neutrophils magnetic isolation

The separation of mouse neutrophils was performed according to the Magnetic Activated Cell Sorting (MACS) protocol provided (Mouse Neutrophil Isolation Kit, Miltenyi, #130-097-658). In short, single cells were obtained from PB, BM or lung tissue. 50 μl Neutrophil Biotin-Antibody Cocktail was added as primary antibodies per 200 μl cell suspension (5 × 10^7^ total cells in MACS buffer) and incubate for 15 min in 4 °C. After washing twice, 100 µl Anti-Biotin MicroBeads was added per 400 μl cell suspension. LS column and MidiMACS separator (Miltenyi) were applied for subsequent magnetic sorting.

The separation of human neutrophils was performed according to the procedure provided (EasySep™ Direct Human Neutrophil Isolation Kit, STEMCELL, #19666). Briefly, isolation cocktail (50 μl/mL) and RapidSpheres™ beads (50 μl/mL) was added into whole blood and incubated for 5 min at room temperature. Then added isolation buffer up to 10 mL, and placed the tube into the EasySep™ Magnets for 5 min. The enriched cell suspension was collected in a new tube and incubate with RapidSpheres ™ beads for 5 min. A second separation with EASYSEP™ Magnets for 5 min was operated and the enriched cell suspension was collected for subsequent in vitro induction.

### Primary tissue culture and supernatant collection

Primary tumor tissue was cut into small piece using sterile ophthalmic scissors. Then samples were put in a 6-well plate with FBS-free RPMI-1640 medium. Culture supernatant was harvested after 24 h and centrifuged at 300 × *g*, for 5 min. The tumor tissue culture supernatant was used for neutrophils induction in vitro. Tumor samples were further digested in medium containing 1 mg/mL collagenase IV and single cell suspension was obtained for apoptosis detection (AnnexinV-FITC/PI apoptosis detection kit, BD Biosciences, #556547).

### Primary tumor and metastatic tumor burden calculation

Tumor burden was calculated as long diameter times short diameter for a single tumor, and burden of multiple lung metastases was added together.

### Tissue hypoxia detection

To detect primary tumor hypoxia, Hypoxyprobe™ Kit was applied (Hypoxyprobe Inc, #HP1). Hypoxyprobe™-1 (pimonidazole HCl) solution (60 mg/kg) was injected (i.p.) into 2 weeks 4T1-bearing mice and primary tumors were resected 1 h later. The tumor tissues were then fixed and embedded. Tissue sections were obtained and stained with mAb1, then with Alexa 594-conjugated secondary reagent.

### Cell surface marker staining and flow cytometry

The isolated single cells from primary tumor, lung, PB or BM were washed with cell staining buffer for twice and then resuspend with 100 μl of cell staining buffer (Biolegend, #420201). Then fluorochrome-conjugated anti-mouse monoclonal antibodies (mAbs) specific for CD45 (Clone 30-F11), CD11b (Clone M1/70), Ly-6G (Clone 1A8), CD62L (Clone MEL-14), TLR2 (Clone T2.5), TLR4 (Clone SA15-21), EpCAM (Clone G8.8), and anti-human mAbs specific for CD45 (Clone HI30), CD11b (Clone ICRF44), CD66b (Clone G10F5), CD62L (Clone DREG-56) were added according to the different experimental requirements. Samples were incubated in dark for 20 min in 4 °C, and washed with cell staining buffer for twice. The mAbs mentioned above were purchased from Biolegend. For flow cytometry analysis, samples were resuspended in 300 μl of cell staining buffer. Data was acquired with a FACSCanto II flow cytometer (BD Biosciences) and analyzed with FlowJo software (V10.0 for Windows). For fluorescence-activated cell sorting (FACS), single cell suspension was sorted with a FACSAria III cell sorter (BD Biosciences). The cell sorting strategy was as follows: 1. CD45-APC/Cy7, CD11b-PE/Cy7, Ly6G-APC, CD62L-PE; 2. EpCAM-PE, CD45-APC/Cy7.

### RNA isolation and quantitative real-time-PCR

Mouse tissues were ground in ceramic mortar containing liquid nitrogen, and total RNA of tissue samples was extracted with TRIzol reagent (Invitrogen, #15596-018). Cell samples were added directly to TRlzol according to the manufacturer’s instructions. Concentration of purified RNA was tested by NanoDrop (Thermo Fisher). 1 μg total RNA was reverse-transcribed into cDNA using PrimeScript™ RT Master Mix (TaKaRa, #RR036A), amplified by TB Green Premix Ex Taq (TaKaRa, #RR420A) and detected by the 7500 Fast Real-Time system (Applied Biosystems). Data were processed using 7500 (V2.3) software (Applied Biosystems). Results were normalized based on housekeeping gene *β-actin* and then expressed as fold upregulation comparing with control.

### Nuclear and cytoplasmic protein extraction

The nucleoprotein and plasma protein were separated using Nuclear and Cytoplasmic Protein Extraction Kit (Beyotime, #P0027) as the manufacturer’s instructions described. Briefly, for cultured cell, the cells were digested with trypsin and washed with PBS, and then 200 μl Reagent A with 1 mM PMSF were added. High speed vortexing for 5 s and ice bath for 15 min were needed. then 10 μl Reagent B was added and the liquid was vortexed for 5 s in high speed with 1 min ice bath. After vortexing for 5 s and centrifugation at 16,000 × *g* for 5 min, the supernatant was collected as cytoplasmic protein. The precipitation was further deal with Nuclear protein extraction reagent with PMSF. After vortexing and ice bath in turn for 30 min, liquid was centrifugated at 16,000 × *g* for 5 min at 4 °C. the supernatant was collected as nuclear protein.

For tissue extraction, fresh tissues were obtained and put on ice immediately. Then the tissue was weighed and cut into piece. 60 mg tissue with 200 μl Reagent A, 10 μl Reagent B and 1 mM PMSF were mixed and glass tissue homogenizer was applied for further grinding. Homogenate was put on the ice for 15 min and centrifuged 1500 × *g*, 5 min, 4 °C. The supernatant was collected as tissue cytoplasmic protein for further concentration detection and HMGB1 content detection.

### Western blot

Total proteins were harvested from sorted neutrophils or cultured tumor cells or fresh frozen tissues and lysed by pre-cooled lysis buffer with a cocktail of protease and phosphatase inhibitor (Thermo Fisher, #78445). Protein concentrations were measured by a bicinchoninic acid (BCA) assay kit (Thermo Fisher, #23227). The proteins were separated by sodium dodecyl sulfate-polyacrylamide gel electrophoresis (SDS-PAGE) and transferred onto polyvinylidene difluoride (PVDF) membrane (Bio-Rad). After blocked by the 5% (w/v) fat-free milk (BD Biosciences, #232100) at room temperature for 1 h, we incubated the membrane with the corresponding primary antibodies overnight at 4 °C followed by the appropriate horseradish peroxidase (HRP)-conjugated secondary antibodies. Immunoreactive bands were identified using enhanced chemiluminescence (Thermo Fisher Pierce, #32109). primary antibody including anti-HMGB1 (1:250, Abcam, #ab79823), anti-β-actin (1:2000, HuaBio, #EM21002), anti-GAPDH (1:2000, HuaBio, #ET1601-4), anti-β-tubulin (1:2000, HuaBio, #EM1602-4), anti-TLR2 (1:1000, Abcam, #ab209217), anti-MyD88 (1:1000, Proteintech, #23230-1-AP), anti-histone H3 (1:2000, HuaBio, #ET1701-64) was applied. Secondary antibodies, including anti-mouse (1:5000, HuaBio, #G1006-1) and anti-rabbit (1:5000, HuaBio, #HA1001) were applied.

### Immunocytofluorescence (ICF)

Tumor cells were plated on sterile round glasses in 12 wells and incubated in nomoxia or hypoxia incubator for indicated times (24, 48, and 72 h). For another, sorted neutrophils subsets were seeded on Poly-D-lysine (0.1 mg/mL, Beyotime, #C0312) coated sterile round glasses at 12 wells in complete RPMI-1640 medium for 1–3 h (depending on the adhesion degree via optical microscope). For destroy NETs, DNAseI (Roche, #11284932001, 1000 Unit/mL) were added (Fig. S6C). Then the glass was fixed with 4% Paraformaldehyde (PFA) for 10 min at room temperature. Cell permeabilization was obtained after 20 min incubation with PBS containing 0.2% TritonX-100 (Sigma-Aldrich, #X100) and blocking by 3% BSA (MP Biomedicals, #0218054990) for 60 min. Cells were incubated with primary antibodies in 4 °C overnight and fluorescent secondary antibody was added then on the cells in 4 °C for 2 h. After washing, DAPI (Invitrogen, #D1306) was added and covered with glass. Samples were analyzed with LSM 710 confocal microscope (Carl Zeiss, Germany). Primary antibodies including HMGB1 (1:250, Abcam, #ab79823), neutrophil elatase (1:200, Abcam, #ab68672), Alpha Tubulin- Alexa Fluor® 488 (1:1000, Abcam, #ab7291) and Sytox Orange Nucleic Acid Stain (Thermo Fisher, #S11368) were applied.

### Immunohistochemistry (IHC) and immunohistofluorescence (IHF)

Mouse tissues for H&E staining or immunohistochemistry was obtained and covered with 4% PFA for at least 24 h, then embedding by paraffin. Immunohistochemistry was performed using standard protocol (Absin, #abs9211). Briefly, 4–5 μm paraffin sections were deparaffinized through alcohol gradients and rehydrated to water. Antigenic retrieval was performed using Tris-EDTA (pH = 9) buffer in thermostatic bath at 98°for 30 minutes. Antibodies specific to HMGB1 (1:350, Abcam, #ab79823) were used in this study.

The human and mouse tumor tissue sections were reviewed and scored by individual researchers (Z. Wang and C.H. Yang). Slides were scanned using Pannoramic MIDI (3DHISTECH Ltd) and images were captured through Pannoramic Viewer software (3DHISTECH Ltd).

Immunohistochemical staining scoring was semi-quantitatively evaluated by staining location, intensity, and the percentage of positive cells. If HMGB1 stained only in cell nucleus, the score was regarded as 1. The plasma staining intensity was graded as 0 (negative), 2 (weak staining), 3 (moderate staining) or 4 (strong staining). The percentage positivity was graded as 1 (<30%), 2 (30–60%), 3 (>60%). The two grades were added together to yield the immunoreactive score (IRS). Cases with discrepancies in IRS were discussed with other pathologists until consensus was reached. Evaluation of immunohistochemical staining was carried out by two researchers (Z. Wang and C.H. Yang) blinded to the clinicopathological characteristics.

### Enzyme-linked immunosorbent assays (ELISA)

HMGB1 concentration was tested in sandwich ELISA. Sample including murine serum, cell culture supernatants were tested by mouse HMGB1 ELISA kit (ArigoBio, #ARG81310) and human serum were tested by human HMGB1 ELISA kit (ArigoBio, #ARG81185). Procedure are operated as the manual protocol provided.

### In vitro neutrophil intervention assay

Neutrophils were isolated from naive BALB/c mice BM and human PB. Cells were suspended in complete RPMI-1640 medium and seeded in plate bottom 96 wells (1 × 10^5^ cells/well). The following reagents/additives were used for in vitro neutrophils intervention. (1) concentrated tissue and cell culture supernatant obtained by ultrafiltration tube centrifugation at 3000 × *g* for 15 min in Fig. 4d, e (Millipore, #UFC900396) (2) TLR agonist including Pam3CSK4 (10 ng/mL, TLR2/TLR1 agonist, InvivoGen, #tlrl-pms), FSL-1(10 ng/mL, TLR2/6 agonist, InvivoGen, #tlrl-fsl), Poly(I:C) (LMW) (100 ng/ml, TLR3 agonist, InvivoGen, #tlrl-picw), MPLA-SM (1 μg/mL, TLR4 agonist, InvivoGen, #tlrl-mpla), FLA-ST (1 μg/mL, TLR5 agonist, InvivoGen, #tlrl-stfla), R848 (1 μg/mL, TLR7/8 agonist, InvivoGen, #tlrl-r848), TLR9 Agonist Kit containing ODN 1585, ODN 1826, ODN 2395 (1 μg/mL, TLR9 agonist, InvivoGen, #tlrl-kit9m) in Fig. 6c; (3) D-Ribose (mixture of isomers, RAGE agonist, MCE, #HY-W018772) in Fig. S5D; (4) O-Vanillin (100 μM, TLR1/2 & TLR2/6 inhibitor, Sigma-Aldrich, #120804) in Fig. 6d; (5) TAK242 (10 µM, TLR4 inhibitor, MCE, #HY-11109) in Fig. 6d; (6) TTP488 (4 nM, RAGE inhibitor, Selleck, #S6415) in Fig. 6d; (7) recombinant murine HMGB1 protein (Biolegend, #764004) in Fig. 4c and recombinant human HMGB1 protein (Biolegend, #557804) in Fig. 5d; (8) HMGB1 inhibitor, including glycyrrhizic acid (200 μM, Sigma-Aldrich, #PHR1516) and ethyl pyruvate (1 mM, Sigma-Aldrich, #E47808) were added (Fig. 4f). (9) 20% human serum including healthy donor, TNBC and non-TNBC in Fig. 5e; Neutrophils were harvested 4 h later for flow cytometry analysis or RT-PCR.

### In vivo recombinant HMGB1 pre-intervention

In order to evaluate the role of recombinant murine HMGB1 protein (rHMGB1) in 4T1 tumor-bearing mice, we conducted experiments as shown in Fig. S6B. Age-matched BALB/c mice were randomly divided into two groups. After using PBS (Control group) or rHMGB1 (rHMGB1 pretreatment) (125 μg/kg (i.v.) every other day) for one week in advance, the mice were injected with 4T1 cells in situ, and then continued to intervene for 2 weeks. The mice were sacrificed 3 weeks after tumor inoculation, and lung tissues were taken for H&E staining.

### Tumor cell metastasis in vivo assay

Short-term observation group (Fig. 2i): to evaluate 4T1 and EMT6 tumor cell form lung metastasis ability, 4T1-Control, 4T1-shHMGB1 and EMT6-Control, EMT6-HMGB1 tumor cell was inoculated in the female BALB/c mice right forth breast pad (1 × 10^5^/100 μl). then luciferase-expressing EMT6 (1 × 10^6^/100 μl) were injected (i.v.) after breast pad inoculation for 2 weeks and in vivo imaging was performed in 2 h.

Long-term observation group: to further evaluate the function of rHMGB1 and CD62L^dim^ neutrophils in lung metastasis formation, experimental grouping was showed as Figs. 7a and S6B. Briefly, luciferase-expressing EMT6 was injected (i.v.) (1 × 10^6^/100 μl) two weeks after EMT6 or 4T1 orthotopic implant, then rHMGB1 (125 μg/kg) or CD62L^dim^ neutrophils obtained from 4T1 2-week tumor-bearing mice’s PB (1 × 10^7^ cells/100 μl) were injected (i.v.) every other day for 2 weeks, as PBS was given meanwhile for control. Mice were injected D-luciferin (i.p.) (Promega, #E1601, 100 mg/kg) and suffered gas anesthesia with isoflurane (RWD, #R510-22) then in vivo bioluminescence imaging were operated using IVIS Lumina LT (Perkin Elmer) at 4-week.

### In vivo therapeutic intervention

For in vivo imaging, luciferase-expressing 4T1 (1 × 10^6^ cells/100 μl) were injected (i.v.) at 2 weeks after 4T1 tumor breast pad inoculation (1 × 10^5^ cells/100 μl), then interventions were lasted for 2 weeks. The intervention strategy was described in Fig. 7b: glycyrrhizic acid (20 mg/kg), ethyl pyruvate (100 mg/kg), O-vanillin (50 mg/kg) were injected (i.p.) and DNAseI (Destroy NETs, Roche, #11284932001, 2.5 mg/kg) was injected (*i.v*.) every other day. mice were injected D-luciferin (i.p.) and suffered gas anesthesia with isoflurane then in vivo bioluminescence imaging were operated using IVIS Lumina LT.

For flow cytometry analysis, 4T1 tumor breast pad inoculation and the intervention were started simultaneously. Glycyrrhizic acid (20 mg/kg), Ethyl pyruvate (100 mg/kg), O-Vanillin (50 mg/kg) were injected (*i.p*.) and DNAseI (2.5 mg/kg) was injected (i.v.) every other day. After 2 weeks, lung tissue and PB were obtained for subsequently flow cytometry analysis.

### Rescue assay

Aged matched BALB/c mice were randomly grouped into 4 groups: 1) 4T1-shHMGB1 group, 2) 4T1-shHMGB1 with rHMGB1 intervention group, 3) 4T1-shHMGB1-HMGB1 overexpression (4T1-shH-H) group, 4) 4T1-shH-H with Ethyl pyruvate (EP) intervention group. For in vivo imaging, luciferase-expressing 4T1 (1 × 10^6^ cells/100 μl) were injected (i.v.) at 2 weeks after different tumor cells inoculation (1 × 10^5^ cells/100 μl). Then EP (100 mg/kg) and rHMGB1 (125 μg/kg) were injected (i.v.) every other day. Interventions were lasted for 2 weeks, and the other two groups were given PBS meanwhile for control mice were injected D-luciferin (i.p.) and suffered gas anesthesia with isoflurane then in vivo bioluminescence imaging were operated using IVIS Lumina LT.

For flow cytometry analysis, different tumor breast pad inoculation and the intervention were started simultaneously. After 2 weeks, lung tissue and PB were obtained for subsequently flow cytometry analysis.

### Statistics

Statistical analysis was performed using Graphpad Prism (V6.0) software. Statistical significance (*p ≤ 0.05, **p ≤ 0.01, and ***p ≤ 0.001) between the means of a minimum of three groups was determined using unpaired two-tailed Student’s *t* test, two-way ANOVA. Results are expressed as the mean value ± SD. All results are representative of at least three independent experiments.

## Supplementary information

Supplementary Figure and Table Legends

Supplementary Figure 1

Supplementary Figure 2

Supplementary Figure 3

Supplementary Figure 4

Supplementary Figure 5

Supplementary Figure 6

Supplementary Table 1

Supplementary Table 2

Supplementary Table 3

Authorship sign
