# Supplementary material for: Tumor-derived HMGB1 induces CD62Ldim neutrophil polarization and promotes lung metastasis in triple-negative breast cancer
Source: Oncogenesis. 2020 Sep 17;9(9):82. doi: 10.1038/s41389-020-00267-x (PMC7499196; doi:10.1038/s41389-020-00267-x)
Supplement: Supplementary file 1 — Supplementary Figure and Table Legends [file 41389_2020_267_MOESM1_ESM.doc]

**Supplementary Figure Legends**

**Figure S1, related to Figure 1.**

(A) KMplot online correlation analysis of HMGB1 expression with overall survival (OS) and Relapse-free survival (RFS) in various cancer patients.

(B) GEO database correlation analysis of HMGB1 expression with OS, RFS and distance metastasis free survival (DMFS) in total breast cancer.

(C) TCGA database and METABRIC database analysis of HMGB1 expression in different molecular types of breast cancer patients.

(D) Immunohistochemical staining scoring was semi-quanti-tatively evaluated by HMGB1 localization, staining intensity and the percentage of positive cells. Specific description in methodology.

(E to G) The HMGB1 staining scores in different molecular types (E) tumor stages (F) and lymph node metastasis (G) of breast cancer patients.

Data are mean± SEM of one representative experiments. Similar results were seen in three independent experiments. Unpaired Student’s t tests, ns, not significant. *p˂0.05, **p˂0.01, ***p˂0.001.

**Figure S2, related to Figure 2.**

(A and B) mRNA expression (A) and protein level (B) of HMGB1 in primary tumor with tumor progression.

(C) Western blot analysis of HMGB1 on EpCAM^+^ cells (regarded as tumor cells) and CD45^+^cells (regarded as immune cells) from primary tumor of 2-week tumor bearing mice.

(D) Elisa analysis of HMGB1 concentration of cultured supernatant from EpCAM^+^ cells and CD45^+^cells from 2-week tumor bearing mice primary tumor.

(E) Flow-cytometry gate strategy (up) and the purity verifies (down) of sorting primary tumor infiltrating EpCAM^+^ cells and CD45^+^cells.

(F) Proliferation of different tumor cell lines *in vitro by* CCK8 analysis.

(G and H) Representative image (G) and quantification (H) of primary tumors and spleens of 2-week tumor bearing mice formed by different tumor cell lines inoculation.

(I and J) The protein level (I) and the mRNA level (J) of HMGB1 in different primary tumor of 2-week tumor bearing mice.

(K and L) Elisa analysis of HMGB1 concentration of tumor tissue cultured supernatant (TTCS) (K) and serum (L) of 2-week tumor bearing mice after different tumor cell lines inoculation.

(M) Pro-metastatic associated genes expression in the lung of 2-week tumor bearing mice.

Data are mean± SEM of one representative experiments. Similar results were seen in three independent experiments. Unpaired Student’s t tests, ns, not significant. *p˂0.05, **p˂0.01, ***p˂0.001.

**Figure S3, related to Figure 3.**

(A) Representative images on HP (Hypoxyprobe™) stained primary tumor sections of 2-week tumor bearing mice.

(B) Western blot analysis of HMGB1 in 4T1 cell line under normoxia and hypoxia for 72h.

(C) mRNA expression of HMGB1 in 4T1 cell line under normoxic and varying degrees of hypoxic conditions.

(D) The survival analysis of the tumor cells under normoxia and hypoxia.

**Figure S4, related to Figure 4 and 5.**

(A to C) Flow-cytometry gate strategy (A) and representative analysis of neutrophils (B) and CD62L^dim^ neutrophils (C) distribution in BM, PB and lung of 2-week tumor bearing mice formed by different tumor cell lines inoculation.

(D) Flow analysis (left) and the quantification (right) of the survival of tumor cells before and after cultured for 24h in 2-week tumor tissue.

(E) Flow-cytometry gate strategy of human PB neutrophils.

Data are mean± SEM of one representative experiments. Similar results were seen in three independent experiments. Unpaired Student’s t tests, ns, not significant. *p˂0.05, **p˂0.01, ***p˂0.001.

**Figure S5, related to Figure 6.**

(A) MFI of TLR4 expression on lung infiltrating neutrophils of naïve mice and 2-week tumor bearing mice by flow cytometry.

(B) Western blot analysis of TLR2 and Myd88 on lung infiltrating CD62L^dim^ neutrophils and CD62L^hi^ neutrophils of tumor bearing mice.

(C) MFI of TLR4 expression on lung infiltrating CD62L^dim^ neutrophils and CD62L^hi^ neutrophils of 2-week tumor bearing mice by flow cytometry.

(D) Quantification of CD62L expression on neutrophils from BM neutrophils of naïve mice treated with the RAGE agonist (D-Ribose) for 4 hours *in vitro*.

(E) Cell immunofluorescent analysis of NETs of BM neutrophils from Balb/c naïve mice treated with or without the TLR2 agonist (FSL-1) or rHMGB1.

(F) Cell immunofluorescent analysis of NETs of BM neutrophils from different naïve mice treated with rHMGB1.

Data are mean± SEM of one representative experiments. Similar results were seen in three independent experiments. Unpaired Student’s t tests, ns, not significant. *p˂0.05, **p˂0.01, ***p˂0.001.

**Figure S6, related to Figure 7.**

(A) Schematic illustration of drug intervention of Figure 7A.

(B) Schematic illustration (left), representative image (middle) and quantification (right) of metastatic lung of 3-week tumor bearing mice pretreated with or without rHMGB1.

(C) Cell immunofluorescent analysis of NETs of lung infiltrating CD62L^dim^ neutrophils from tumor bearing mice with or without DNAseⅠ treatment.

(D) The protein level of HMGB1 in 4T1 cell lines. shH, *HMGB1* knockout 4T1 cell line; shH-H, *HMGB1* overexpressing shHMGB1 4T1 cell line.

(E) The protein level (left) and the mRNA level (right) of HMGB1 in primary tumor with different tumor cell lines inoculation.

Data are mean± SEM of one representative experiments. Similar results were seen in three independent experiments. Unpaired Student’s t tests, ns, not significant. *p˂0.05, **p˂0.01, ***p˂0.001.

**Supplementary Table Legends**

**Supplementary Table 1. Clinico-pathological characteristics of patients included in this study (Fig. 1D).**

**Supplementary Table 2. Clinico-pathological characteristics of patients included in this study (Fig. 1E)**

**Supplementary Table 3. Clinico-pathological characteristics of patients included in this study (Fig. 1F)**
