# Supplementary figures and images for: Tumor-derived HMGB1 induces CD62Ldim neutrophil polarization and promotes lung metastasis in triple-negative breast cancer
Source: Oncogenesis. 2020 Sep 17;9(9):82. doi: 10.1038/s41389-020-00267-x (PMC7499196; doi:10.1038/s41389-020-00267-x)

Figure S1, related to Figure 1.

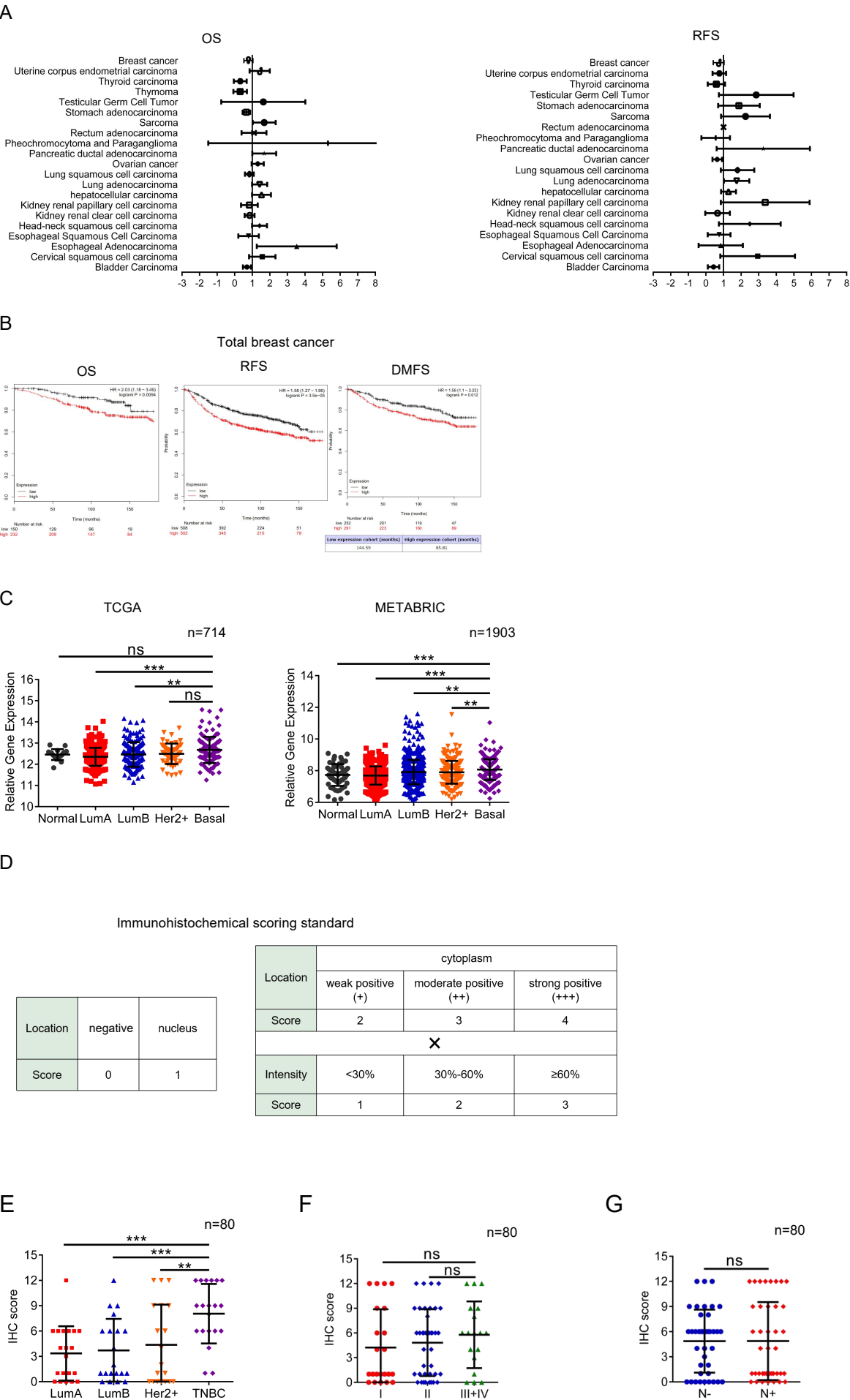

Supplement: Supplementary file 2 — Supplementary Figure 1 [file 41389_2020_267_MOESM2_ESM.pdf]

Figure S2, related to Figure 2.

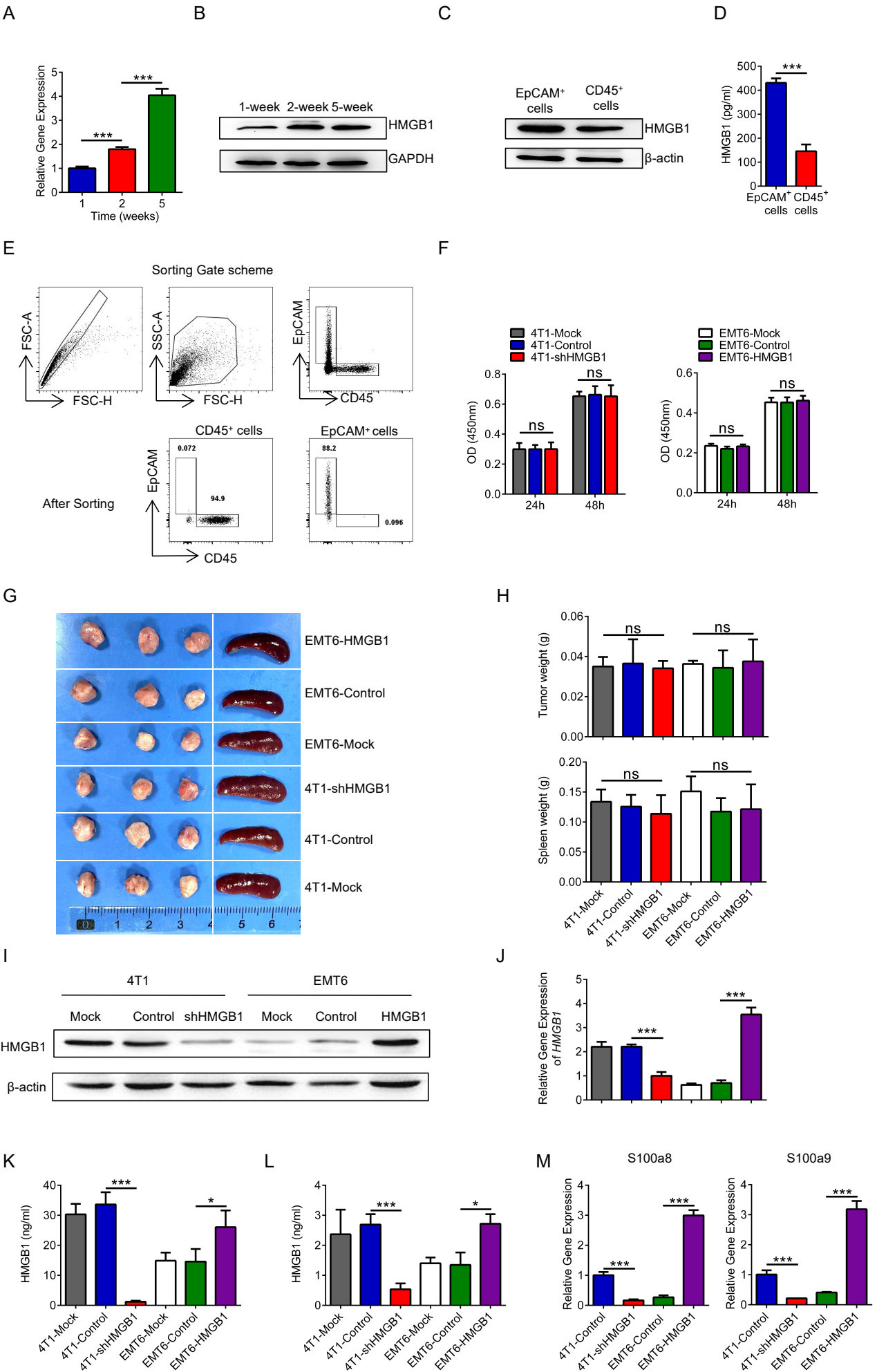

Supplement: Supplementary file 3 — Supplementary Figure 2 [file 41389_2020_267_MOESM3_ESM.pdf]

Figure S3, related to Figure 3.

A

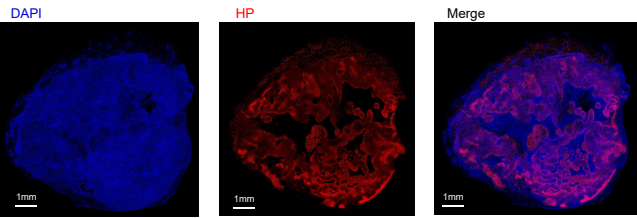

B

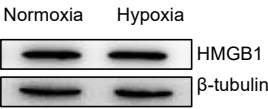

C

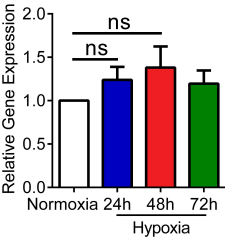

D

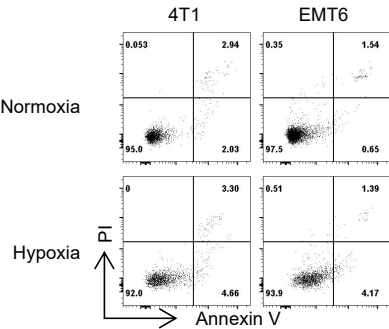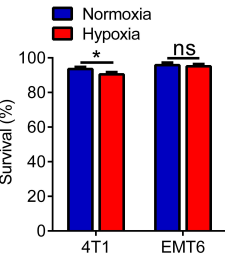

Supplement: Supplementary file 4 — Supplementary Figure 3 [file 41389_2020_267_MOESM4_ESM.pdf]

**Figure S4, related to Figure 4 and 5.**

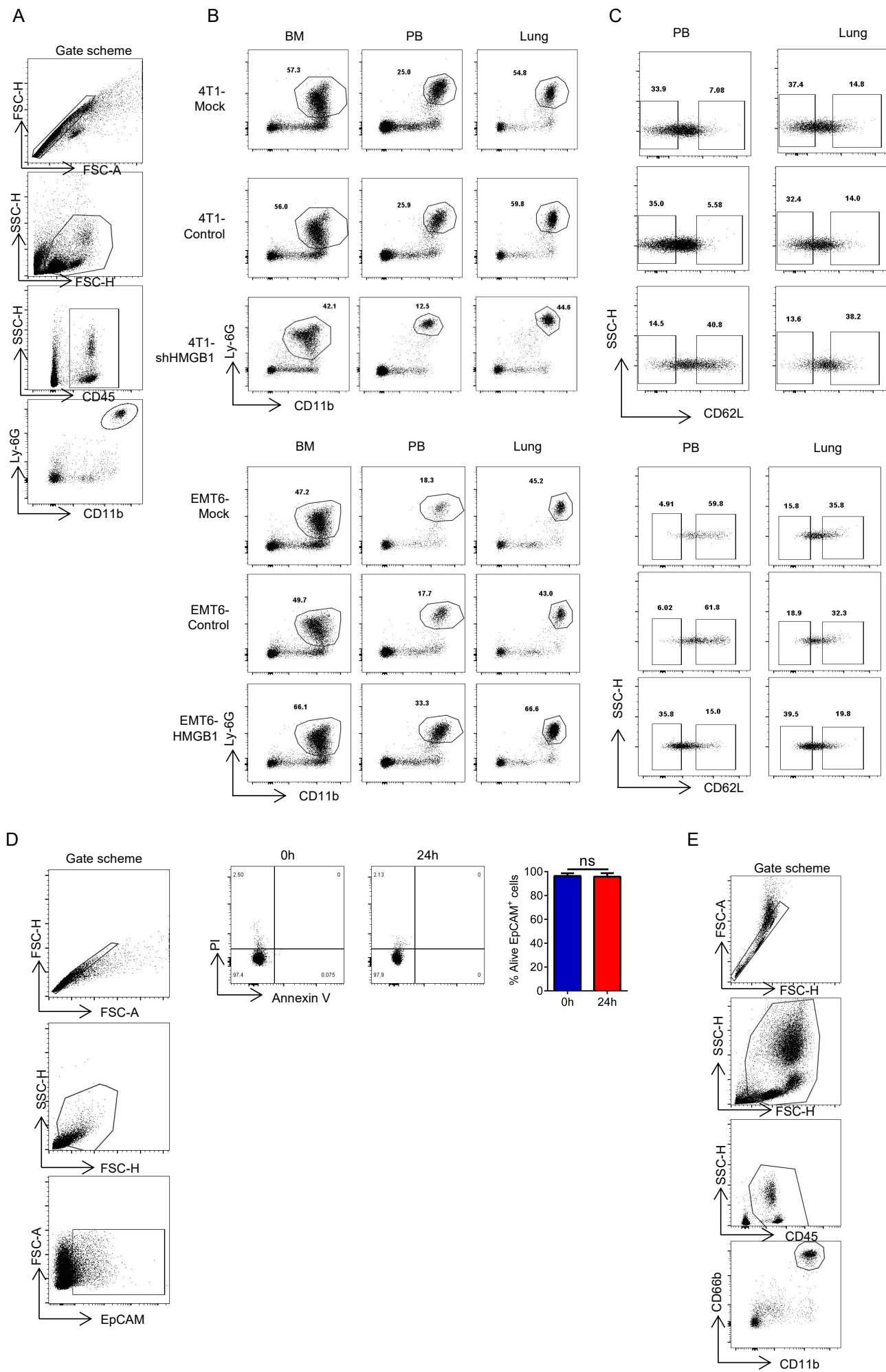

Supplement: Supplementary file 5 — Supplementary Figure 4 [file 41389_2020_267_MOESM5_ESM.pdf]

Figure S5, related to Figure 6.

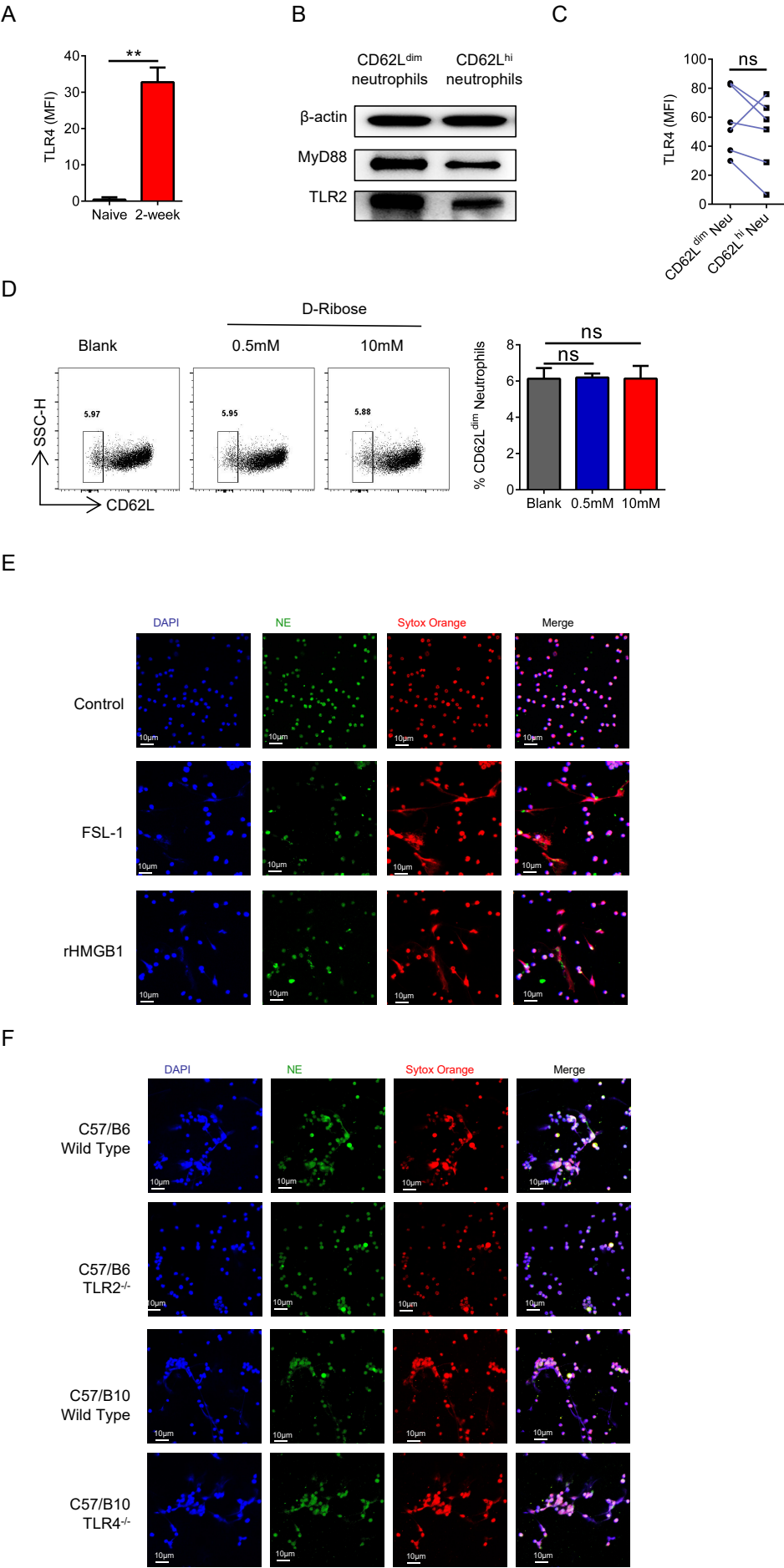

Supplement: Supplementary file 6 — Supplementary Figure 5 [file 41389_2020_267_MOESM6_ESM.pdf]

Figure S6, related to Figure 7.

A

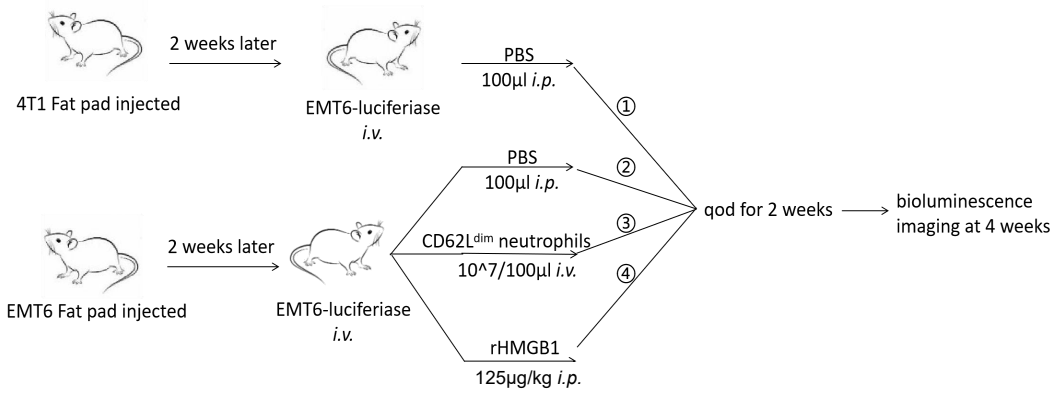

B

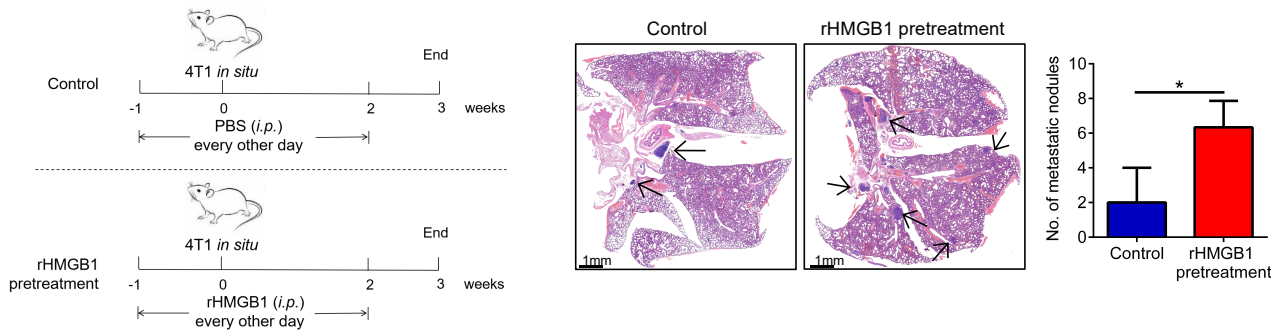

C

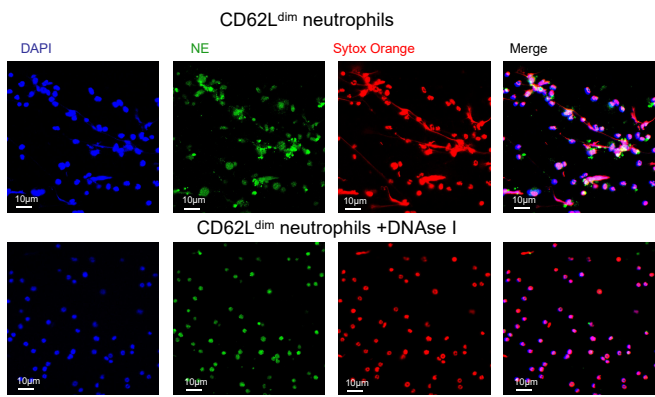

D

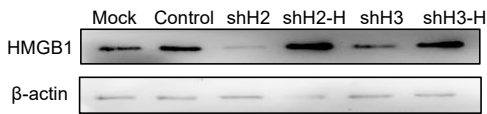

E

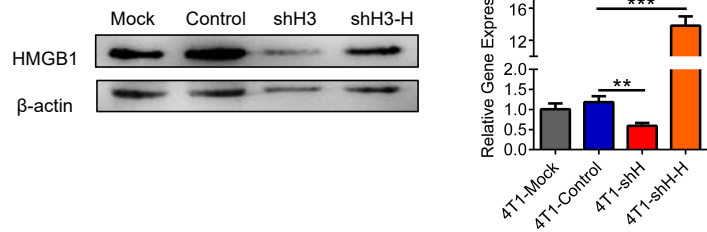

Supplement: Supplementary file 7 — Supplementary Figure 6 [file 41389_2020_267_MOESM7_ESM.pdf]
