# Supplementary material for: Tumor-derived HMGB1 induces CD62Ldim neutrophil polarization and promotes lung metastasis in triple-negative breast cancer
Source: Oncogenesis. 2020 Sep 17;9(9):82. doi: 10.1038/s41389-020-00267-x (PMC7499196; doi:10.1038/s41389-020-00267-x)
Supplement: Supplementary file 8 — Supplementary Table 1 [file 41389_2020_267_MOESM8_ESM.doc]

| **Supplementary Table 1. Clinico-pathological characteristics of patients included in this study (Fig. 1D).** | | | | | | | | | | | | |
| --- | --- | --- | --- | --- | --- | --- | --- | --- | --- | --- | --- | --- |
| **Patient ID** | **Sex** | **Age(y)** | **Diagnosis** | **ER** | **PR** | **HER2** | **Ki67** | **Histological classification** | **T stage** | **N stage** | **M stage** | **pathological TNM** |
| BC1 | F | 57 | breast cancer | - | - | + | 20% | HER2 | 2 | 1 | 0 | IIB |
| BC2 | F | 71 | breast cancer | - | - | + | 30% | HER2 | 2 | 2 | 0 | IIIA |
| BC3 | F | 69 | breast cancer | - | - | + | 30% | HER2 | 1 | 0 | 0 | IA |
| BC4 | F | 48 | breast cancer | - | - | + | 40% | HER2 | 1 | 1 | 0 | IIA |
| BC5 | F | 52 | breast cancer | - | - | + | 20% | HER2 | 2 | 0 | 0 | IIA |
| BC6 | F | 59 | breast cancer | - | - | + | 60% | HER2 | 1 | 0 | 0 | IA |
| BC7 | F | 62 | breast cancer | - | - | + | 20% | HER2 | 2 | 0 | 0 | IIA |
| BC8 | F | 38 | breast cancer | - | - | + | 20% | HER2 | 4 | 0 | 0 | IIIB |
| BC9 | F | 53 | breast cancer | - | - | + | 70% | HER2 | 1 | 0 | 0 | IA |
| BC10 | F | 57 | breast cancer | - | - | + | 30% | HER2 | 2 | 1 | 0 | IIB |
| BC11 | F | 67 | breast cancer | - | - | + | 40% | HER2 | 1 | 2 | 0 | IIIA |
| BC12 | F | 61 | breast cancer | - | - | + | 80% | HER2 | 2 | 2 | 0 | IIIA |
| BC13 | F | 57 | breast cancer | - | - | + | 20% | HER2 | 1 | 1 | 0 | IIA |
| BC14 | F | 54 | breast cancer | - | - | + | 10% | HER2 | 2 | 0 | 0 | IIA |
| BC15 | F | 51 | breast cancer | - | - | + | 80% | HER2 | 1 | 0 | 0 | IA |
| BC16 | F | 57 | breast cancer | - | - | + | 15% | HER2 | 1 | 0 | 0 | IA |
| BC17 | F | 62 | breast cancer | - | - | + | 30% | HER2 | 2 | 1 | 0 | IIB |
| BC18 | F | 57 | breast cancer | - | - | + | 40% | HER2 | 1 | 1 | 0 | IIA |
| BC19 | F | 62 | breast cancer | - | - | + | 15% | HER2 | 1 | 1 | 0 | IIA |
| BC20 | F | 64 | breast cancer | - | - | + | 20% | HER2 | 2 | 0 | 0 | IIA |
| BC21 | F | 48 | breast cancer | + | + | - | 10% | Luminal A | 3 | 1 | 0 | IIIA |
| BC22 | F | 62 | breast cancer | + | - | - | 5% | Luminal A | 1 | 0 | 0 | IA |
| BC23 | F | 90 | breast cancer | + | + | - | 5% | Luminal A | 2 | 0 | 0 | IIA |
| BC24 | F | 65 | breast cancer | + | + | - | 10% | Luminal A | 2 | 0 | 0 | IIA |
| BC25 | F | 69 | breast cancer | + | + | - | 5% | Luminal A | 1 | 0 | 0 | IA |
| BC26 | F | 64 | breast cancer | + | - | - | 5% | Luminal A | 2 | 0 | 0 | IIA |
| BC27 | F | 79 | breast cancer | + | + | - | 10% | Luminal A | 1 | 0 | 0 | IA |
| BC28 | F | 33 | breast cancer | + | + | - | 10% | Luminal A | 1 | 2 | 0 | IIIA |
| BC29 | F | 72 | breast cancer | + | + | - | 5% | Luminal A | 2 | 0 | 0 | IIA |
| BC30 | F | 55 | breast cancer | + | + | - | 0% | Luminal A | 1 | 1 | 0 | IIA |
| BC31 | F | 49 | breast cancer | + | + | - | 10% | Luminal A | 1 | 1 | 0 | IIA |
| BC32 | F | 46 | breast cancer | + | - | - | 15% | Luminal A | 1 | 0 | 0 | IA |
| BC33 | F | 48 | breast cancer | + | + | - | 10% | Luminal A | 1 | 1 | 0 | IIA |
| BC34 | F | 50 | breast cancer | + | + | - | 15% | Luminal A | 1 | 0 | 0 | IA |
| BC35 | F | 53 | breast cancer | + | + | - | 5% | Luminal A | 1 | 1 | 0 | IIA |
| BC36 | F | 71 | breast cancer | + | + | - | 5% | Luminal A | 1 | 2 | 0 | IIIA |
| BC37 | F | 63 | breast cancer | + | - | - | 10% | Luminal A | 2 | 0 | 0 | IIA |
| BC38 | F | 71 | breast cancer | + | + | - | 10% | Luminal A | 2 | 1 | 0 | IIB |
| BC39 | F | 75 | breast cancer | + | + | - | 5% | Luminal A | 0 | 1 | 0 | IIA |
| BC40 | F | 50 | breast cancer | + | - | - | 5% | Luminal A | 0 | 2 | 0 | IIIA |
| BC41 | F | 68 | breast cancer | + | + | + | 20% | Luminal B | 2 | 2 | 0 | IIIA |
| BC42 | F | 65 | breast cancer | + | - | - | 15% | Luminal B | 1 | 0 | 0 | IA |
| BC43 | F | 46 | breast cancer | + | + | + | 30% | Luminal B | 1 | 0 | 0 | IA |
| BC44 | F | 64 | breast cancer | + | - | + | 10% | Luminal B | 1 | 1 | 0 | IIA |
| BC45 | F | 71 | breast cancer | + | - | + | 5% | Luminal B | 1 | 0 | 0 | IA |
| BC46 | F | 49 | breast cancer | + | + | + | 20% | Luminal B | 2 | 2 | 0 | IIIA |
| BC47 | F | 56 | breast cancer | + | + | - | 80% | Luminal B | 2 | 2 | 0 | IIIA |
| BC48 | F | 67 | breast cancer | + | - | + | 10% | Luminal B | 2 | 1 | 0 | IIB |
| BC49 | F | 38 | breast cancer | + | + | - | 15% | Luminal B | 2 | 1 | 0 | IIB |
| BC50 | F | 66 | breast cancer | + | + | + | 40% | Luminal B | 2 | 2 | 0 | IIIA |
| BC51 | F | 55 | breast cancer | + | + | - | 30% | Luminal B | 1 | 0 | 0 | IA |
| BC52 | F | 54 | breast cancer | + | + | - | 20% | Luminal B | 1 | 0 | 0 | IA |
| BC53 | F | 68 | breast cancer | + | + | + | 20% | Luminal B | 1 | 0 | 0 | IA |
| BC54 | F | 75 | breast cancer | + | - | - | 30% | Luminal B | 2 | 0 | 0 | IIA |
| BC55 | F | 63 | breast cancer | + | + | + | 20% | Luminal B | 1 | 0 | 0 | IA |
| BC56 | F | 72 | breast cancer | + | - | + | 20% | Luminal B | 2 | 0 | 0 | IIA |
| BC57 | F | 65 | breast cancer | + | + | - | 50% | Luminal B | 2 | 0 | 0 | IIA |
| BC58 | F | 36 | breast cancer | + | - | + | 30% | Luminal B | 1 | 1 | 0 | IIA |
| BC59 | F | 50 | breast cancer | + | + | - | 40% | Luminal B | 2 | 1 | 0 | IIB |
| BC60 | F | 53 | breast cancer | + | + | + | 15% | Luminal B | 2 | 2 | 0 | IIIA |
| BC61 | F | 53 | breast cancer | - | - | - | 20% | TNBC | 1 | 1 | 0 | IIA |
| BC62 | F | 40 | breast cancer | - | - | - | 70% | TNBC | 1 | 0 | 0 | IA |
| BC63 | F | 44 | breast cancer | - | - | - | 40% | TNBC | 1 | 3 | 0 | IV |
| BC64 | F | 51 | breast cancer | - | - | - | 70% | TNBC | 2 | 0 | 1 | IIA |
| BC65 | F | 50 | breast cancer | - | - | - | 70% | TNBC | 2 | 0 | 0 | IIA |
| BC66 | F | 51 | breast cancer | - | - | - | 15% | TNBC | 1 | 0 | 0 | IA |
| BC67 | F | 62 | breast cancer | - | - | - | 30% | TNBC | 1 | 0 | 0 | IA |
| BC68 | F | 38 | breast cancer | - | - | - | 10% | TNBC | 1 | 1 | 0 | IIA |
| BC69 | F | 35 | breast cancer | - | - | - | 70% | TNBC | 2 | 0 | 0 | IIA |
| BC70 | F | 74 | breast cancer | - | - | - | 30% | TNBC | 2 | 0 | 0 | IIA |
| BC71 | F | 52 | breast cancer | - | - | - | 60% | TNBC | 1 | 1 | 0 | IIA |
| BC72 | F | 32 | breast cancer | - | - | - | 40% | TNBC | 1 | 0 | 0 | IA |
| BC73 | F | 76 | breast cancer | - | - | - | 5% | TNBC | 3 | 3 | 0 | IIIC |
| BC74 | F | 64 | breast cancer | - | - | - | 60% | TNBC | 2 | 3 | 0 | IIIC |
| BC75 | F | 81 | breast cancer | - | - | - | 40% | TNBC | 2 | 2 | 0 | IIIA |
| BC76 | F | 30 | breast cancer | - | - | - | 90% | TNBC | 2 | 2 | 0 | IIIA |
| BC77 | F | 50 | breast cancer | - | - | - | 40% | TNBC | 2 | 0 | 0 | IIA |
| BC78 | F | 63 | breast cancer | - | - | - | 70% | TNBC | 1 | 1 | 0 | IIA |
| BC79 | F | 38 | breast cancer | - | - | - | 80% | TNBC | 1 | 0 | 0 | IA |
| BC80 | F | 64 | breast cancer | - | - | - | 30% | TNBC | 0 | 1 | 0 | IIA |
