# Supplementary material for: Tumor-derived HMGB1 induces CD62Ldim neutrophil polarization and promotes lung metastasis in triple-negative breast cancer
Source: Oncogenesis. 2020 Sep 17;9(9):82. doi: 10.1038/s41389-020-00267-x (PMC7499196; doi:10.1038/s41389-020-00267-x)
Supplement: Supplementary file 9 — Supplementary Table 2 [file 41389_2020_267_MOESM9_ESM.doc]

| **Supplementary Table 2. Clinico-pathological characteristics of patients included in this study (Fig. 1E)** | | | | | | | | | | | | | |
| --- | --- | --- | --- | --- | --- | --- | --- | --- | --- | --- | --- | --- | --- |
| **Patient ID** | **Sex** | **Age(y)** | **Surgery** | **Diagnosis** | **ER** | **PR** | **HER2** | **Ki67** | **Histological classification** | **T stage** | **N stage** | **M stage** | **Metastasis within 10 years** |
| BC81 | F | 45 | 2002 | breast cancer | - | - | - | 30% | TNBC | 3 | 0 | 0 | Y |
| BC82 | F | 51 | 2004 | breast cancer | - | - | - | 50% | TNBC | 2 | 1 | 0 | Y |
| BC83 | F | 34 | 1999 | breast cancer | - | + | - | 10% | Luminal A | 1 | 2 | 0 | Y |
| BC84 | F | 75 | 2006 | breast cancer | - | - | - | 10% | TNBC | 1 | 0 | 0 | Y |
| BC85 | F | 57 | 2007 | breast cancer | - | - | + | 0% | HER2 | 1 | 1 | 0 | Y |
| BC86 | F | 48 | 2006 | breast cancer | - | - | - | 70% | TNBC | 2 | 2 | 0 | Y |
| BC87 | F | 51 | 2008 | breast cancer | - | - | - | 30% | TNBC | 2 | 0 | 0 | Y |
| BC88 | F | 50 | 2009 | breast cancer | - | - | + | 40% | HER2 | 1 | 1 | 0 | Y |
| BC89 | F | 40 | 2009 | breast cancer | - | - | - | 40% | TNBC | 3 | 2 | 0 | Y |
| BC90 | F | 54 | 2010 | breast cancer | - | - | - | 50% | TNBC | 3 | 1 | 0 | Y |
| BC91 | F | 84 | 2012 | breast cancer | - | - | - | 10% | TNBC | 3 | 2 | 0 | Y |
| BC92 | F | 46 | 2010 | breast cancer | - | - | + | 50% | HER2 | 4 | 2 | 0 | Y |
| BC93 | F | 47 | 2010 | breast cancer | - | - | - | 40% | TNBC | 3 | 1 | 0 | Y |
| BC94 | F | 53 | 2012 | breast cancer | + | - | - | 20% | Luminal B | 2 | 0 | 0 | Y |
| BC95 | F | 43 | 2013 | breast cancer | - | - | + | 20% | HER2 | 3 | 3 | 0 | Y |
| BC96 | F | 42 | 2011 | breast cancer | - | - | - | 10% | TNBC | 2 | 0 | 0 | Y |
| BC97 | F | 30 | 2015 | breast cancer | - | - | - | 30% | TNBC | 3 | 2 | 0 | Y |
| BC98 | F | 48 | 2012 | breast cancer | - | - | + | 60% | HER2 | 2 | 1 | 0 | Y |
| BC99 | F | 26 | 2014 | breast cancer | - | - | - | 20% | TNBC | 1 | 2 | 0 | Y |
| BC100 | F | 43 | 2013 | breast cancer | - | - | - | 40% | TNBC | 1 | 1 | 0 | Y |
| BC101 | F | 60 | 2015 | breast cancer | - | - | + | 20% | Luminal B | 2 | 0 | 0 | Y |
| BC102 | F | 56 | 2005 | breast cancer | + | - | + | 50% | Luminal B | 2 | 2 | 0 | N |
| BC103 | F | 54 | 2005 | breast cancer | - | - | + | 30% | HER2 | 3 | 1 | 0 | N |
| BC104 | F | 35 | 2007 | breast cancer | - | - | + | 30% | HER2 | 2 | 1 | 0 | N |
| BC105 | F | 45 | 2009 | breast cancer | + | + | - | 30% | Luminal B | 1 | 1 | 0 | N |
| BC106 | F | 59 | 2005 | breast cancer | + | + | - | 20% | Luminal B | 3 | 0 | 0 | N |
| BC107 | F | 49 | 2007 | breast cancer | + | + | + | 40% | Luminal B | 2 | 1 | 0 | N |
| BC108 | F | 48 | 2009 | breast cancer | + | + | + | 10% | Luminal B | 2 | 1 | 0 | N |
| BC109 | F | 35 | 2009 | breast cancer | + | + | + | 10% | Luminal B | 3 | 3 | 0 | N |
| BC110 | F | 49 | 2005 | breast cancer | + | + | + | 40% | Luminal B | 2 | 2 | 0 | N |
| BC111 | F | 52 | 2009 | breast cancer | - | - | + | 10% | HER2 | 1 | 2 | 0 | N |
| BC112 | F | 51 | 2009 | breast cancer | + | + | + | 10% | Luminal B | 1 | 2 | 0 | N |
| BC113 | F | 56 | 2009 | breast cancer | + | + | + | 30% | Luminal B | 3 | 2 | 0 | N |
| BC114 | F | 49 | 2009 | breast cancer | + | + | + | 30% | Luminal B | 3 | 1 | 0 | N |
| BC115 | F | 36 | 2009 | breast cancer | - | - | + | 10% | HER2 | 1 | 1 | 0 | N |
| BC116 | F | 49 | 2009 | breast cancer | - | - | + | 40% | HER2 | 1 | 0 | 0 | N |
| BC117 | F | 38 | 2009 | breast cancer | + | + | + | 60% | Luminal B | 3 | 2 | 0 | N |
| BC118 | F | 48 | 2009 | breast cancer | - | + | + | 80% | Luminal B | 2 | 2 | 0 | N |
| BC119 | F | 39 | 2009 | breast cancer | + | - | - | 10% | Luminal A | 2 | 3 | 0 | N |
| BC120 | F | 39 | 2007 | breast cancer | - | + | - | 30% | Luminal B | 3 | 2 | 0 | N |
| BC121 | F | 43 | 2005 | breast cancer | - | - | + | 10% | HER2 | 2 | 2 | 0 | N |
| BC122 | F | 46 | 2010 | breast cancer | + | + | - | 5% | Luminal A | 3 | 2 | 0 | N |
