# Supplementary material for: Tumor-derived HMGB1 induces CD62Ldim neutrophil polarization and promotes lung metastasis in triple-negative breast cancer
Source: Oncogenesis. 2020 Sep 17;9(9):82. doi: 10.1038/s41389-020-00267-x (PMC7499196; doi:10.1038/s41389-020-00267-x)
Supplement: Supplementary file 10 — Supplementary Table 3 [file 41389_2020_267_MOESM10_ESM.doc]

| **Supplementary Table 3. Clinico-pathological characteristics of patients included in this study (Fig. 1F)** | | | | | | | | | | | |
| --- | --- | --- | --- | --- | --- | --- | --- | --- | --- | --- | --- |
| **Patient ID** | **Sex** | **Age(y)** | **Diagnosis** | **ER** | **PR** | **HER2** | **Ki67** | **Histological classification** | **T stage** | **N stage** | **M stage** |
| BC123 | F | 59 | breast cancer | - | - | + | 50% | HER2 | 1 | 0 | 0 |
| BC124 | F | 45 | breast cancer | - | - | - | 80% | TNBC | 1 | 2 | 0 |
| BC125 | F | 51 | breast cancer | + | + | - | 30% | Luminal B | 2 | 1 | 0 |
| BC126 | F | 43 | breast cancer | + | + | - | 30% | Luminal B | 1 | 0 | 0 |
| BC127 | F | 41 | breast cancer | - | - | - | 70% | TNBC | 2 | 0 | 0 |
| BC128 | F | 41 | breast cancer | + | + | + | 10-20% | Luminal B | 2 | 1 | 0 |
| BC129 | F | 60 | breast cancer | - | - | - | 0% | TNBC | 1 | 2 | 0 |
| BC130 | F | 43 | breast cancer | + | + | - | 5% | Luminal B | 2 | 0 | 0 |
| BC131 | F | 47 | breast cancer | - | - | - | 0% | TNBC | 1 | 2 | 0 |
| BC132 | F | 57 | breast cancer | - | - | - | 0% | TNBC | 1 | 1 | 0 |
| BC133 | F | 40 | breast cancer | + | + | - | 80% | Luminal B | 1 | 1 | 0 |
| BC134 | F | 53 | breast cancer | - | - | + | 20% | HER2 | 2 | 1 | 1 |
| BC135 | F | 41 | breast cancer | - | - | + | 20% | HER2 | 1 | 0 | 0 |
| BC136 | F | 49 | breast cancer | + | + | - | 10% | Luminal A | 2 | 0 | 0 |
| BC137 | F | 49 | breast cancer | + | + | - | 40% | Luminal B | 1 | 0 | 0 |
| BC138 | F | 47 | breast cancer | + | + | + | 20% | Luminal B | 2 | 0 | 0 |
| BC139 | F | 51 | breast cancer | + | + | - | 20% | Luminal B | 2 | 1 | 0 |
| BC140 | F | 35 | breast cancer | - | - | - | 50% | TNBC | 2 | 1 | 0 |
| BC141 | F | 32 | breast cancer | - | - | + | 30% | HER2 | 3 | 1 | 0 |
| BC142 | F | 31 | breast cancer | + | + | + | 30% | Luminal B | 1 | 0 | 0 |
| BC143 | F | 55 | breast cancer | - | - | - | 20% | TNBC | 2 | 1 | 0 |
| BC144 | F | 46 | breast cancer | + | + | - | 40% | Luminal B | 2 | 0 | 0 |
| BC145 | F | 56 | breast cancer | + | + | - | 80% | Luminal B | 1 | 1 | 0 |
| BC146 | F | 41 | breast cancer | + | + | - | 5% | Luminal A | 2 | 1 | 0 |
| BC147 | F | 38 | breast cancer | + | + | - | 20% | Luminal B | 3 | 2 | 0 |
| BC148 | F | 72 | breast cancer | + | + | - | 50% | Luminal B | 1 | 0 | 0 |
| BC149 | F | 46 | breast cancer | + | - | + | 40% | Luminal B | 1 | 0 | 0 |
| BC150 | F | 48 | breast cancer | - | - | - | 40% | TNBC | 3 | 2 | 1 |
| BC151 | F | 42 | breast cancer | + | + | - | 5% | Luminal A | 1 | 0 | 0 |
| BC152 | F | 55 | breast cancer | + | + | - | 10% | Luminal A | 2 | 0 | 0 |
| BC153 | F | 47 | breast cancer | + | + | - | 30% | Luminal B | 1 | 0 | 0 |
| BC154 | F | 55 | breast cancer | + | + | - | 15% | Luminal A | 1 | 1 | 0 |
| BC155 | F | 58 | breast cancer | + | + | - | 5-10% | Luminal A | 2 | 1 | 0 |
| BC156 | F | 47 | breast cancer | + | - | + | 10% | Luminal A | 1 | 1 | 0 |
| BC157 | F | 60 | breast cancer | + | + | - | 10% | Luminal A | 4 | 1 | 1 |
| BC158 | F | 45 | breast cancer | - | - | - | 70% | TNBC | 2 | 2 | 0 |
| BC159 | F | 45 | breast cancer | + | + | - | 10% | Luminal A | 1 | 0 | 0 |
| BC160 | F | 32 | breast cancer | - | - | - | 50% | TNBC | 2 | 1 | 0 |
| BC161 | F | 76 | breast cancer | - | - | - | 40% | Luminal B | 1 | 1 | 0 |
| BC162 | F | 72 | breast cancer | - | - | + | 20% | HER2 | 1 | 0 | 0 |
| BC163 | F | 45 | breast cancer | + | - | - | 10% | Luminal A | 1 | 1 | 0 |
| BC164 | F | 43 | breast cancer | + | + | - | 10% | Luminal A | 2 | 0 | 0 |
| BC165 | F | 49 | breast cancer | - | + | - | 10% | Luminal A | 1 | 0 | 0 |
| BC166 | F | 45 | breast cancer | + | - | - | 60% | Luminal B | 2 | 2 | 0 |
| BC167 | F | 49 | breast cancer | - | - | + | 10% | HER2 | 2 | 0 | 0 |
| BC168 | F | 53 | breast cancer | - | - | + | 10% | HER2 | 2 | 1 | 0 |
| BC169 | F | 55 | breast cancer | - | - | + | 15% | HER2 | 2 | 0 | 0 |
| BC170 | F | 43 | breast cancer | + | + | - | 30% | Luminal B | 2 | 0 | 0 |
| BC171 | F | 50 | breast cancer | - | - | + | 40% | HER2 | 2 | 0 | 0 |
| BC172 | F | 39 | breast cancer | - | - | + | 50% | HER2 | 2 | 1 | 0 |
| BC173 | F | 57 | breast cancer | + | + | - | 60% | Luminal B | 1 | 1 | 0 |
| BC174 | F | 47 | breast cancer | + | + | + | 60% | Luminal B | 3 | 1 | 0 |
| BC175 | F | 54 | breast cancer | + | + | - | 50% | Luminal B | 1 | 0 | 0 |
| BC176 | F | 61 | breast cancer | + | + | - | 30% | Luminal B | 1 | 1 | 0 |
| BC177 | F | 62 | breast cancer | + | + | - | 30% | Luminal B | 2 | 1 | 0 |
| BC178 | F | 47 | breast cancer | + | + | + | 30% | Luminal B | 1 | 1 | 0 |
| BC179 | F | 73 | breast cancer | - | - | + | 70% | HER2 | 2 | 1 | 0 |
| BC180 | F | 50 | breast cancer | + | + | - | 10% | Luminal A | 1 | 1 | 0 |
| BC181 | F | 78 | breast cancer | - | - | + | 70% | HER2 | 2 | 1 | 0 |
| BC182 | F | 47 | breast cancer | + | + | - | 10% | Luminal A | 1 | 0 | 0 |
| BC183 | F | 69 | breast cancer | - | - | + | 20% | HER2 | 2 | 1 | 0 |
| BC184 | F | 58 | breast cancer | + | + | + | 50% | Luminal B | 1 | 0 | 0 |
| BC185 | F | 43 | breast cancer | - | - | + | 15% | Luminal B | 2 | 0 | 0 |
| BC186 | F | 55 | breast cancer | + | - | + | 30% | Luminal B | 3 | 0 | 0 |
| BC187 | F | 48 | breast cancer | + | - | + | 40% | Luminal B | 1 | 0 | 0 |
| BC188 | F | 57 | breast cancer | + | + | - | 45% | Luminal B | 1 | 0 | 0 |
| BC189 | F | 53 | breast cancer | + | + | - | 40% | Luminal B | 1 | 1 | 0 |
| BC190 | F | 50 | breast cancer | - | - | + | 45% | Luminal B | 2 | 1 | 0 |
| BC191 | F | 48 | breast cancer | + | + | - | 30% | Luminal B | 3 | 1 | 0 |
| BC192 | F | 35 | breast cancer | + | + | + | 60% | Luminal B | 2 | 2 | 0 |
| BC193 | F | 56 | breast cancer | + | + | - | 30% | Luminal B | 2 | 1 | 0 |
| BC194 | F | 40 | breast cancer | + | + | - | 40% | Luminal B | 1 | 0 | 0 |
| BC195 | F | 60 | breast cancer | + | + | - | 10% | Luminal A | 1 | 1 | 0 |
| BC196 | F | 59 | breast cancer | + | + | + | 40% | Luminal B | 1 | 0 | 0 |
| BC197 | F | 63 | breast cancer | - | - | + | 80% | HER2 | 2 | 1 | 0 |
| BC198 | F | 68 | breast cancer | + | + | - | 5% | Luminal A | 2 | 0 | 0 |
| BC199 | F | 75 | breast cancer | + | + | - | 10% | Luminal A | 1 | 1 | 0 |
| BC200 | F | 75 | breast cancer | + | + | - | 80% | Luminal B | 2 | 1 | 0 |
| BC201 | F | 56 | breast cancer | - | + | + | 30% | Luminal B | 2 | 1 | 0 |
| BC202 | F | 56 | breast cancer | + | - | - | 30% | Luminal B | 1 | 1 | 0 |
| BC203 | F | 53 | breast cancer | - | - | + | 5% | HER2 | 1 | 0 | 0 |
| BC204 | F | 39 | breast cancer | + | + | - | 10% | Luminal A | 1 | 1 | 0 |
| BC205 | F | 55 | breast cancer | + | - | + | 30% | Luminal B | 2 | 1 | 0 |
| BC206 | F | 67 | breast cancer | - | - | + | 30-40% | HER2 | 3 | 2 | 0 |
| BC207 | F | 50 | breast cancer | + | - | + | 20% | Luminal B | 1 | 2 | 0 |
| BC208 | F | 60 | breast cancer | + | + | - | 10% | Luminal A | 1 | 0 | 0 |
