# Supplementary material for: Tumor-derived HMGB1 induces CD62Ldim neutrophil polarization and promotes lung metastasis in triple-negative breast cancer
Source: Oncogenesis. 2020 Sep 17;9(9):82. doi: 10.1038/s41389-020-00267-x (PMC7499196; doi:10.1038/s41389-020-00267-x)
Supplement: Supplementary file 11 — Authorship sign [file 41389_2020_267_MOESM11_ESM.pdf]

SPRINGER NATURE

In accordance with Springer Nature Authorship Policy we agree to change the authors of the manuscript as indicated below.

NAME OF JOURNAL: Oncogenesis

TITLE OF MANUSCRIPT: Tumor-derived HMGB1 induces CD62Ldim neutrophil polarization and promotes lung metastasis in triple-negative breast cancer

MANUSCRIPT NUMBER: ONCSIS-20-0214

CORRESPONDING AUTHORS NAME: Jian Huang

PREVIOUS AUTHOR NAMES:

Zhen Wang ,Chenghui Yang, Lili Li, Xiaoyan Jin, Zhigang Zhang, Haiyan Zheng, Jun Pan , Zhou Jiang, Ke Su, Baizhou Li, Xuan Shao, Fuming Qiu, Jun Yan, Jian Huang

UPDATED AUTHOR NAMES:

Zhen Wang ,Chenghui Yang, Lili Li, Xiaoyan Jin, Zhigang Zhang, Haiyan Zheng, Jun Pan, Liyun Shi, Zhou Jiang, Ke Su, Baizhou Li, Xuan Shao, Fuming Qiu, Jun Yan, Jian Huang

CHANGE TO AUTHOR LIST:

Liyun Shi

Print Name	Signature	Date
Zhen Wang	Zhen Wang	2020. 7. 14
Chenghui Yang	Chenghui Yang	2020.7.14
Lili Li	Lili Li	2020.7.14
Xiaoyan Jin	Xiaoyan Jin	2020.7.14
Zhigang Zhang	Zhigang Zhang	2020. 7.14

Haiyan Zheng	Haiyan Zheng	2020.7.14
Jun Pan	Jun Pan	2020.7.14
Liyun Shi	Liyun Shi	2020.7.14
Zhou Jiang	Zhou Jiang	2020.7.14
Ke Su	Ke Su	2020.7.14
Baizhou Li	Baizhou Li	2020.7.14
Xuan Shao	Xuan Shao	2020.7.14
Fuming Qiu	Fuming Qiu	2020.7.14
Jun Yan	[illegible]	2020.7.14
Jian Huang	[illegible]	2020.7.14
